# Buprenorphine exposure levels to optimize treatment outcomes in opioid use disorder

**DOI:** 10.3389/fphar.2022.1052113

**Published:** 2022-11-18

**Authors:** Celine M. Laffont, Eliford Ngaimisi, Mathangi Gopalakrishnan, Vijay Ivaturi, Malcolm Young, Mark K. Greenwald, Christian Heidbreder

**Affiliations:** ^1^ Indivior Inc., North Chesterfield, VA, United States; ^2^ Center for Translational Medicine, University of Maryland, Baltimore, MD, United States; ^3^ Department of Psychiatry and Behavioral Neurosciences, Wayne State University School of Medicine, Detroit, MI, United States

**Keywords:** (extended-release) buprenorphine, opioid blockade, withdrawal, craving, exposure-response, opioid use disorder, optimized treatment outcome

## Abstract

The severity of the ongoing opioid crisis, recently exacerbated by the COVID-19 pandemic, emphasizes the importance for individuals suffering from opioid use disorder (OUD) to have access to and receive efficacious, evidence-based treatments. Optimal treatment of OUD should aim at blocking the effects of illicit opioids while controlling opioid craving and withdrawal to facilitate abstinence from opioid use and promote recovery. The present work analyses the relationship between buprenorphine plasma exposure and clinical efficacy in participants with moderate to severe OUD using data from two clinical studies (39 and 504 participants). Leveraging data from placebo-controlled measures assessing opioid blockade, craving, withdrawal and abstinence, we found that buprenorphine plasma concentrations sustained at 2–3 ng/ml (corresponding to ≥70% brain *mu*-opioid receptor occupancy) optimized treatment outcomes in the majority of participants, while some individuals (e.g., injecting opioid users) needed higher concentrations. Our work also included non-linear mixed effects modeling and survival analysis, which identified a number of demographic, genetic and social factors modulating treatment response and retention. Altogether, these findings provide key information on buprenorphine plasma levels that optimize clinical outcomes and increase the likelihood of individual treatment success. NLM identifiers: NCT02044094, NCT02357901.

## 1 Introduction

Opioid use disorder (OUD) is characterized by the repeated seeking or use of an opioid despite adverse social, psychological and physical consequences (American Psychiatric Association, 2013). In the U.S., this chronic, relapsing disease has reached epidemic proportion and was declared a public health emergency in 2017. In recent years the epidemic has been fueled by potent synthetic opioids (fentanyl and analogues), often surreptitiously mixed with heroin, which have become the main driver of opioid overdose deaths (Ochalek et al., 2019; Jones et al., 2020). Recent adjusted estimates suggest past-year OUD affected 7,6 million Americans aged 12 or older in 2019, with approximately 86.6% of those not receiving treatment for OUD (Krawczyk et al., 2022). Lately, the opioid crisis has been compounded by the coronavirus disease 2019 (COVID-19) pandemic as well as co-use or contamination of psychostimulants with illicitly manufactured fentanyl or heroin (Jones et al., 2020; Niles et al., 2021). Individuals with OUD are at higher risk for COVID-19 and particularly vulnerable to treatment interruption, isolation, and stress, all of which can trigger misuse of prescribed opioids, illicit opioid use and relapse (Rodda et al., 2020; Niles et al., 2021; Wang et al., 2021). Early in the pandemic, drug testing revealed increased positivity rates for non-prescribed fentanyl (+35%), heroin (+44%) and opioids (+10%) (Niles et al., 2021). Since then, multiple U.S. studies have shown increases in opioid-related mortality and emergency department visits (Rodda et al., 2020; Slavova et al., 2020; Holland et al., 2021). The predicted provisional number of opioid overdose deaths in the U.S. increased to 81,991 in the 12-month period ending in December 2021, up from 70,029 in December 2020^1^.

More than ever, it is critical that individuals suffering from OUD receive efficacious, evidence-based treatments. Currently, three medications have been approved in the U.S.: methadone (*mu*-opioid receptor [MOR] full agonist), buprenorphine (MOR partial agonist), and naltrexone (MOR antagonist). Due to its partial agonism, buprenorphine presents a reduced risk of respiratory depression, offering a potential advantage compared with full agonists like methadone (Walsh et al., 1995; ASAM, 2020). Buprenorphine is most frequently dispensed to patients as take-home transmucosal (buccal, sublingual) medication for daily administration. However, this treatment modality presents several limitations, including challenging adherence to treatment (Tkacz et al., 2012; Ronquest et al., 2018), risk of misuse, abuse and diversion (Lofwall and Walsh, 2014; Haight et al., 2019), as well as suboptimal plasma levels resulting in lower occupancy of MORs toward the end of the daily dosing interval (Greenwald et al., 2007).

These limitations can be addressed using long-acting injectable formulations. RBP-6000 or SUBLOCADE (hereafter BUP-XR) is the first monthly buprenorphine injection approved in the U.S. for treatment of moderate-to-severe OUD. BUP-XR was designed and dosing regimens selected to ensure patients are exposed to safe and therapeutic levels throughout the month, with no drop in concentrations that would trigger re-emergence of opioid withdrawal, craving and potential relapse to opioid use (Haight et al., 2019; Andorn et al., 2020). This required a deep understanding of BUP-XR pharmacokinetics as well as relationships linking buprenorphine plasma concentrations, MOR occupancy and pharmacodynamic effects (Nasser et al., 2014; Laffont et al., 2016; Jones et al., 2021).

**FIGURE 1:**
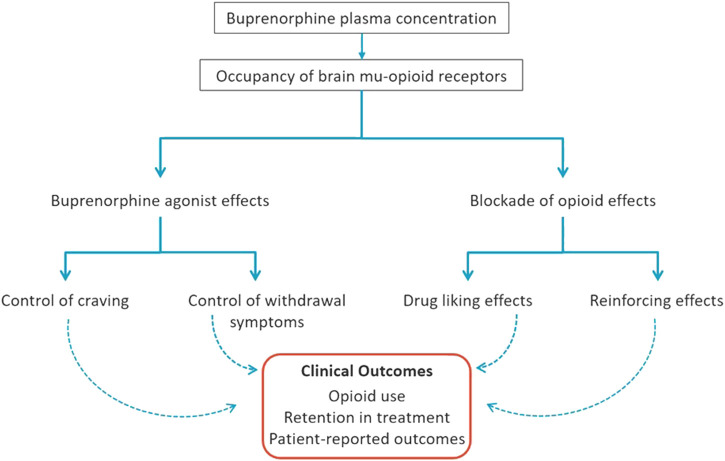
Mechanisms of Buprenorphine Efficacy for Treatment of Opioid Use Disorder. Buprenorphine administration dose-dependently increases buprenorphine plasma concentration and occupancy of brain *mu*-opioid receptors (MORs) that translate into beneficial clinical outcomes. Through MOR occupancy, buprenorphine produces two types of effects: 1) opioid agonist effects in physically-dependent individuals (e.g., those with opioid use disorder) that include attenuation of opioid craving and withdrawal symptoms, and 2) blockade of the agonist effects of exogenous opioids (e.g., heroin, fentanyl) including drug liking and reinforcing effects (seeking/self-administration). Altogether, these agonist and blockade effects of buprenorphine, mediated by brain MORs at optimal concentration levels discussed herein, facilitate physiological stability, reduction in illicit opioid reinforcement and, in combination with other features of treatment, a shift toward natural sources of reinforcement to promote recovery.

In a review of the neurobiology of addiction, Volkow et al. (2016) defined three components of addictive diseases: “Binge/intoxication” related to reward pathways (driven by euphoric and reinforcing effects of the addictive substance), “withdrawal/negative affect” (following withdrawal of the addictive substance), and “preoccupation/anticipation” (commonly referred to as craving). Figure 1 illustrates how buprenorphine can address all components of OUD, *via* its agonist effects (attenuating opioid craving and withdrawal symptoms) and by blocking abuse-related subjective effects (e.g., drug liking) and reinforcing effects (e.g., seeking and self-administration) of the abused opioid(s).

Relationships between buprenorphine plasma concentrations, brain MOR occupancy and effects on disease signs and symptoms were previously examined in two thematically linked laboratory studies conducted in 5 and 10 heroin-dependent subjects (Greenwald et al., 2003; Greenwald et al., 2007). A key finding was that different levels of exposure (i.e., plasma concentrations, MOR occupancy) were needed to control different aspects of the disease. Opioid withdrawal symptoms were controlled when at least 50% of MORs were occupied (plasma concentrations ≥ 1 ng/ml), whereas opioid blockade was achieved when ≥ 70% of MORs were occupied (plasma concentrations ≥ 2–3 ng/ml) (Greenwald et al., 2007; Greenwald et al., 2014; Nasser et al., 2014). In these studies, opioid blockade referred to the ability of buprenorphine to suppress the subjective effects of hydromorphone. These findings were pivotal in identifying target buprenorphine plasma concentrations of ≥ 2 ng/ml that drove the clinical development of BUP-XR.

In the present study, we extend this previous quantitative work by analysing two BUP-XR clinical studies: a Phase 2 study assessing blockade of hydromorphone’s subjective and reinforcing effects, and a Phase 3 double-blind, placebo-controlled efficacy study assessing reductions in opioid use, craving and withdrawal symptoms. Concentration-response analyses were conducted for all clinical measures to refine our understanding of buprenorphine plasma levels (driving MOR occupancy) necessary to maximize buprenorphine efficacy in most patients. We present the results of those concentration-response analyses based on clinical observations and the development of non-linear mixed effects models. Covariate analyses assessed the additional impact of participants’ demographic, genetic, social, and clinical characteristics for key clinical outcomes including opioid use and treatment retention.

## 2 Materials and methods

Data collected from a Phase 2 opioid blockade study (NCT02044094) and a Phase 3 double-blind efficacy study (NCT02357901) of BUP-XR in participants with OUD were used for analysis.

### 2.1 Phase 2 study design

This single-center, open-label study assessed the blockade of opioid subjective and reinforcing effects following treatment with two monthly injections of 300 mg BUP-XR. The study was conducted in 39 male and female participants aged 18–55 years with moderate-to-severe OUD (based on Diagnostic and Statistical Manual of Mental Disorders, Fifth Edition [DSM-5] criteria; American Psychiatric Association, 2013) who were not seeking treatment. Detailed study design and inclusion/exclusion criteria can be found in Nasser et al. (2016). Study participants were compensated for their time on the study.

Prior to receiving BUP-XR, eligible participants were inducted and stabilized on transmucosal buprenorphine over 2 weeks, reaching a final dose between 8 and 24 mg/day. Participants then received a first subcutaneous injection of 300 mg BUP-XR on Day 1 and a second subcutaneous injection of 300 mg BUP-XR on Day 29. Throughout the study, participants were challenged with intramuscular injections of hydromorphone or placebo. Challenge sessions were performed at screening (Day -17 to Day -15), at the end of the transmucosal buprenorphine treatment period (Day -3 to Day -1), and then every week following each BUP-XR injection through 8 weeks after the second BUP-XR injection. Each week, challenges occurred on 3 consecutive residential days. On the morning of each day, participants received either placebo, 6 mg hydromorphone, or 18 mg hydromorphone in a blinded randomized manner. Subjective opioid effects were measured after each challenge using visual analogue scales (VAS) for “Drug Liking” (primary endpoint), “Any Drug Effect”, “Good Drug Effect”, “Bad Drug Effect”, “Sedation” and “High”. Each scale was unipolar, ranging from zero (no effect) to 100 mm (maximal effect). Measurements were done prior to and 15, 30, 45, 60, 75, 90, 120, 150, 180, 210, 240, 270, and 300 min after challenge with hydromorphone or placebo. To be enrolled in the study, participants had to show a positive response to 18 mg hydromorphone at screening, i.e., individuals with a peak drug liking VAS score < 40 mm, or less than a 20-mm (hydromorphone - placebo) difference in drug liking, were not allowed to continue in the study.

Complementary experimental procedures were conducted each afternoon to assess the reinforcing effects of the placebo or hydromorphone challenge dose received that same morning. Those procedures occurred at least 5 h after the morning challenge and consisted of a series of 12 trials. On each trial, the participant could choose to earn $2.00 (value based on Comer et al., 2008; Greenwald and Steinmiller, 2009; Greenwald et al., 2013) or 1/12^th^ unit of the morning dose of hydromorphone or placebo. For each trial, participants had to “mouse”-click repeatedly on a “drug” or “money” icon on a computer screen. The number of clicks to earn each unit of drug or money increased exponentially over trials (from 5 clicks to earn the first unit to 2,160 clicks to earn the 12^th^ unit) according to a progressive ratio schedule of reinforcement detailed in Supplementary Table S1. The increase in the number of clicks occurred independently for drug and money. At the end of the task, the amount of hydromorphone earned was delivered as a single bolus intramuscular injection. Participants who earned only money also received an injection of 0.45% normal saline so that the participant’s choice was not influenced by the attempt to avoid an injection.

Blood samples were taken throughout the study for measurement of buprenorphine plasma concentrations. Samples were collected before and 1.5 h after each transmucosal buprenorphine dose from Day -4 to Day -1, before and 24 h after each BUP-XR injection on Day 1 and Day 29, and prior to each challenge with hydromorphone or placebo. Buprenorphine plasma concentrations were determined using a validated liquid chromatography and tandem mass spectrometric method with a lower limit of quantification of 0.05 ng/ml (Nasser et al., 2016).

### 2.2 Phase 3 study design

The Phase 3 study was a randomized, double-blind, placebo-controlled, multi-center study designed to assess the efficacy, safety and tolerability of multiple subcutaneous injections of BUP-XR. Eligible participants were men and women aged 18–65 years who met DSM-5 criteria for moderate or severe OUD and who were seeking treatment. Participants were not allowed to participate in the study if they had received medication-assisted treatment for OUD within 90 days of enrolment. The average length of reported opioid use was 11–12 years. Detailed study design and inclusion/exclusion criteria can be found in Haight et al. (2019). Participants were compensated for their time on the study.

After screening, participants were inducted and stabilized on transmucosal buprenorphine over 1–2 weeks, reaching a final dose between 8 and 24 mg/day. Eligible participants were then randomized to receive 1) 2 monthly injections of 300 mg BUP-XR followed by 4 monthly injections of 100 mg BUP-XR (300/100 mg dosing regimen; n = 203); 2) 6 monthly injections of 300 mg BUP-XR (300/300 mg dosing regimen; *n* = 201); or 3) 6 monthly injections of volume-matched placebo (*n* = 100). The 100-mg maintenance dose was selected to maintain buprenorphine plasma concentrations achieved with the two initial monthly doses of 300 mg (Jones et al., 2021). Alternatively, the 300-mg maintenance dose provided higher concentrations hypothesized to be needed by some individuals with OUD depending on their drug-use history and clinical condition. After the first injection of BUP-XR or placebo on Day 1, participants were not allowed any supplemental transmucosal buprenorphine except for a 5-day taper (6–2 mg/day) on Days 1–5. Participants who required supplemental transmucosal buprenorphine after Day 1 were to be withdrawn for lack of efficacy and referred for appropriate treatment. All participants received weekly individual drug counselling during the study.

Measures of efficacy included centrally-tested urine drug screens, self-reports of illicit opioid use (recorded on a Timeline Follow Back interview), and assessments on the Opioid Craving VAS, Clinical Opiate Withdrawal Scale (COWS) and Subjective Opiate Withdrawal Scale (SOWS). The Timeline Follow Back interview, administered electronically by an interviewer, was used to assess drug use since the last study visit. Participants reported use or no use (i.e., frequency and amount of use were not captured). Urine drug screens were done with immunoassays to detect opiates, oxycodone, methadone, and buprenorphine among other substances of abuse; buprenorphine was only assessed at screening. Confirmatory testing for opioids was done with gas chromatography combined with mass spectrometry for codeine, hydrocodone, hydromorphone, methadone, morphine, oxycodone and oxymorphone. Additional information on urine drug screen immunoassays and confirmatory testing is provided in Supplementary Table S2. Investigator site staff and participants were blinded to urine drug screen results, except for those performed at screening to assess subject eligibility.

Self-reports and urine drug screens were assessed at screening and every week after each injection of BUP-XR or placebo; a urine drug screen was also performed 24 h after each injection. Scores on the Opioid Craving VAS, COWS and SOWS were obtained at the same times as urine drug screens, with additional measurements performed during induction and stabilization with transmucosal buprenorphine prior to randomization.

Blood samples for determination of buprenorphine plasma concentrations were taken on the last day of transmucosal buprenorphine treatment (Day -1: pre-dose and 1–2 h post-dose) and during the randomized treatment phase (pre-dose and every week after injection, with additional samples at 4 and 24 h post-dose). Plasma concentrations of buprenorphine were determined using the same validated bioanalytical method as in the Phase 2 study.

### 2.3 Analysis of opioid subjective and reinforcing effects

The blockade of hydromorphone subjective effects was evaluated based on drug liking VAS scores in the Phase 2 study (primary endpoint). Analysis of those data was previously published (Nasser et al., 2016); however, it used the mean and not the maximum drug liking score measured over 5 h after each challenge, leading to potential underestimation of hydromorphone effect. Also, that previous analysis used a linear mixed-effects model assuming data were normally distributed, while the distribution of VAS scores was skewed towards zero. We therefore conducted a new and more robust analysis to assess whether the peak drug liking VAS score measured after challenge with 6 mg or 18 mg hydromorphone was “non-inferior” to that measured after challenge with placebo. Medians of peak drug liking VAS scores were calculated for 6 mg and 18 mg hydromorphone after correction with placebo data (i.e., after subtracting the peak VAS score measured on that week’s placebo challenge). Opioid blockade was concluded when the upper bound of the 95% confidence interval of the placebo-corrected median was less than or equal to a non-inferiority margin of 20 mm. This non-inferiority margin was established by the U.S. Food and Drug Administration who had reviewed historical response to opioid agonists in unblocked subjects using a unipolar drug liking VAS^2^.

The relationship between buprenorphine plasma concentration and drug liking response was assessed for each hydromorphone challenge dose. Buprenorphine plasma concentrations were categorized into bins, and the percentage of placebo-corrected peak drug liking scores > 20 mm was calculated for each concentration bin and plotted against buprenorphine levels. Here and elsewhere, fixed bin intervals of 0.5 ng/ml were used, except when the number of observations was low and intervals had to be merged. Intervals were selected to allow a good characterization of the shape of the curve while maintaining sufficient precision for calculating percentages within each bin.

The blockade of hydromorphone reinforcing effects was evaluated based on the number of hydromorphone units that participants chose to earn during afternoon choice sessions. A previous analysis of the same data used breakpoint values, which correspond to the number of mouse clicks achieved to earn the last hydromorphone unit (Nasser et al., 2016). This previous analysis used a mixed-effects model for repeated data under the assumption that breakpoint values were continuous. Here, we used mixed-effects logistic regression to account for the discrete nature of measurements (number of earned hydromorphone dose units). Five ordered categories were defined to achieve sufficient data in each group: zero drug units (participants choosing only money); 1-3 drug units; 4-6 drug units; 7–11 drug units and 12 drug units (participants choosing only hydromorphone). A population pharmacokinetic model (non-linear mixed effects model), describing buprenorphine plasma concentrations after subcutaneous injection of BUP-XR and transmucosal buprenorphine administration, was previously developed from the combined analysis of BUP-XR clinical trials including the Phase 3 efficacy study (Jones et al., 2021). This previous model was used to derive individual predictions of buprenorphine plasma concentration which served as a time-varying covariate in the model.

### 2.4 Analyses of clinical efficacy measures

Opioid use was the primary efficacy measure in the Phase 3 study and was based on the combination of urine drug screen results and self-reported use. The participant’s result was considered negative for opioid use when both the urine drug screen and self-report were negative; otherwise, the result was considered positive. At the 24-h timepoint following each injection, only a urine drug screen was performed, and the result was considered negative when the urine drug screen was negative.

Other measures of efficacy included craving rated on the Opioid Craving VAS, and withdrawal symptoms measured with the COWS and the SOWS. The COWS is an electronic questionnaire with 11 items completed by the clinician (total score between 0 and 48), while the SOWS is a 16-item scale completed by the participant (total score between 0 and 64) (Handelsman et al., 1987; Wesson and Ling, 2003). To assess opioid craving, participants were provided a computerized tablet that displayed a 100-mm line with 0 at the left end and 100 at the right end and asked: “With zero (0) meaning “No Craving At All” and 100 meaning “Strongest Craving Ever” please indicate the point on the line that represents your current state.” This Opioid Craving VAS was recently validated psychometrically on a large OUD patient population to support its use to measure the severity of opioid craving and its ability to predict opioid use (Boyett et al., 2021).

For the purpose of the analyses, Opioid Craving VAS scores were categorized as 0, 1–5, 6–20 and >20 mm (categorization was data-driven). COWS scores were categorized as 5–12 (mild), 13–24 (moderate), 25–36 (moderately severe) and 37–48 (severe) based on Wesson and Ling (2003), with two additional categories (0 and 1–4) to account for the large amount of zero scores reported. SOWS scores were categorized as no withdrawal (0), mild withdrawal (1–10), moderate withdrawal (11–20), moderately severe withdrawal (21–30) and severe withdrawal (31–64).

The relationship between buprenorphine plasma concentration and response was assessed for each efficacy measure. Buprenorphine plasma concentrations were categorized into bins as previously described, and the percentage of observations meeting a defined criterion (e.g., negative opioid use, opioid craving ≤ 5, COWS≤ 4 or SOWS ≤ 10) was calculated for each concentration bin and plotted against buprenorphine levels.

Finally, an integrated model was developed to describe longitudinal measures of opioid use and opioid craving in the Phase 3 study, with a time-to-event model to account for participant dropout. Observed data for opioid use (yes/no) were analysed using a mixed-effects logistic regression model. Observed data for opioid craving (4 ordered categories) were analysed using a mixed-effects logistic regression model for ordinal data. Each model included buprenorphine plasma concentration as a time-varying covariate, based on individual predictions generated with a previous population pharmacokinetic model (Jones et al., 2021). For the modeling of dropout, different distribution functions (constant hazard, Gompertz, Weibull) were tested for the hazard of dropout. There was no noticeable difference in dropout rates between BUP-XR 300/100 mg and 300/300 mg regimens, therefore active vs. placebo treatment was used as a covariate in place of buprenorphine plasma concentration. As indicated in the Results section, dropout was successfully predicted from baseline participant characteristics and recorded measures of efficacy, supporting missing-at-random mechanisms. Consequently, each model could be fitted separately and the combined (integrated) model was used for model evaluation, by comparing observations to simulated data under the study design using prediction-corrected visual predictive checks (Bergstrand et al., 2011).

Covariate analyses were conducted to assess the impact of participants’ characteristics on opioid use, opioid craving and dropout rate. Covariates included demographic factors (age, sex, weight, body mass index, race); baseline clinical characteristics (Beck Depression Inventory, Brief Pain Inventory, Clinical Global Impression-Severity score, use of opioids by injectable vs. non-injectable route); social characteristics (employment, health insurance); and genotypes for opioid receptor subtypes and dopamine D_2_ receptor. Single nucleotide polymorphisms investigated were rs1799971 for *OPRM1* (*mu*-opioid receptor); rs2234918, rs581111 and rs678849 for *OPRD1* (*delta*-opioid receptor); rs1051660 for *OPRK1* (*kappa*-opioid receptor); and rs1800497 for *DRD2* (dopamine D_2_ receptor). For the dropout model, observed measures of opioid use and opioid craving were tested as time-varying covariates using the last-observation-carried-forward (LOCF) approach.

Covariate effects were first evaluated based on exploratory Kaplan-Meier plots (dropout model) or empirical Bayes estimates (EBEs) of individual parameters from the base model (opioid use and opioid craving models). The identification of potential relationships with EBEs was based on visual inspection, statistical testing (Pearson correlation test for continuous covariates; chi-squared test for categorical covariates) and physiological relevance. Covariate relationships identified on EBEs or observed data were further tested by modeling using forward/backward selection with significance levels of 0.05 and 0.01, respectively. For that purpose, an automated stepwise forward inclusion and backward elimination algorithm implemented in PsN (Linearized Stepwise Covariate Model building; Khandelwal et al., 2011; Svensson and Karlsson, 2014) was used.

### 2.5 Software

Models were developed in NONMEM version 7.3 and older (ICON Development Solutions, Ellicott City, MD), which is the reference software for population pharmacokinetic/pharmacodynamic modeling using non-linear mixed effects models. The Laplace method in NONMEM was used for estimation of model parameters. Perl-speaks-NONMEM (PsN) version 4.4.0 was used to operate NONMEM. Other analyses were conducted in R software version 3.3.1 or older.

### 2.6 Study approval

All studies were conducted in accordance with principles and requirements of the International Council for Harmonization Good Clinical Practice guidelines and the principles of the Declaration of Helsinki. Written informed consent was obtained from all participants before starting any study-related procedure. Clinical study protocols, informed consent forms and all other appropriate study-related documents were reviewed and approved by Institutional Review Boards (IRBs) (MidLands IRB for the Phase 2 study; Quorum Review IRB for the Phase 3 study).

## 3 Results

### 3.1 Blockade of opioid subjective and reinforcing effects

**FIGURE 2:**
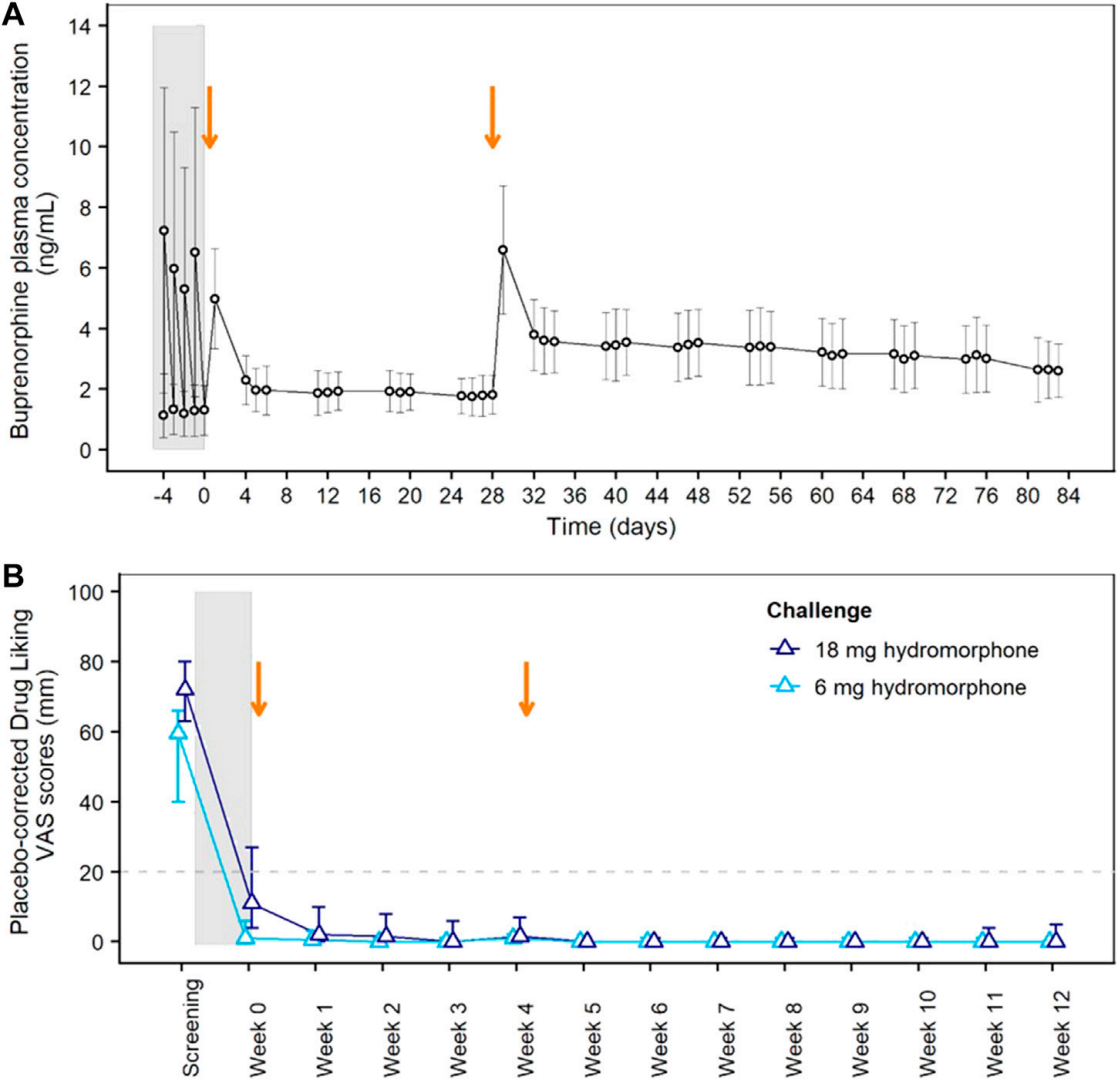
Pharmacokinetics of BUP-XR and Blockade of Hydromorphone Subjective Effects (Drug Liking) in the Phase 2 Study. **(A)** Mean (±SD) buprenorphine plasma concentrations vs. time showing daily fluctuations in buprenorphine plasma concentrations during transmucosal buprenorphine treatment compared to BUP-XR sustained profile. **(B)** Peak drug liking VAS scores after placebo-correction following 6 mg hydromorphone (light blue) or 18 mg hydromorphone (dark blue) challenge. The median placebo-corrected peak VAS score is shown by treatment week, together with its 95% confidence interval (CI; vertical line). Opioid blockade was demonstrated at a given time point when the 95% CI was below the non-inferiority margin of 20 mm (horizontal dashed line). The grey shaded area on both plots indicates the induction and stabilization period with transmucosal buprenorphine (8–24 mg/day) prior to BUP-XR injections; the two vertical arrows represent the two monthly subcutaneous injections of 300 mg BUP-XR. SD: standard deviation; VAS: visual analogue scale.

Blockade of opioid subjective and reinforcing effects was evaluated in the Phase 2 study. Study participant disposition and baseline characteristics are provided in Supplementary Figure S1 and Supplementary Table S3, respectively. Figure 2A shows buprenorphine plasma concentrations during the initial transmucosal buprenorphine treatment, illustrating the daily fluctuations, and following the 2 monthly injections of BUP-XR. Each BUP-XR injection yielded a rapid increase in buprenorphine plasma levels, peaking at 24 h post-dose, then slowly decreasing to a plateau. Buprenorphine plasma concentration during the “plateau” phase was around 2 ng/ml for the first injection and 3 ng/ml for the second injection.

Placebo-corrected “drug liking” scores measured after each hydromorphone challenge are shown in Figure 2B. Whereas hydromorphone liking scores were elevated at screening, non-inferiority analysis showed hydromorphone 6-mg and 18-mg doses were not liked more than placebo throughout the 12-week BUP-XR treatment period, indicating that buprenorphine concentrations reached with BUP-XR injections suppressed hydromorphone liking. Non-inferiority was not observed for 18 mg hydromorphone during transmucosal buprenorphine treatment when plasma concentration averaged 1.2 ng/ml. A tabular presentation of the results is available in the supplemental material (Supplementary Table S4).

**FIGURE 3:**
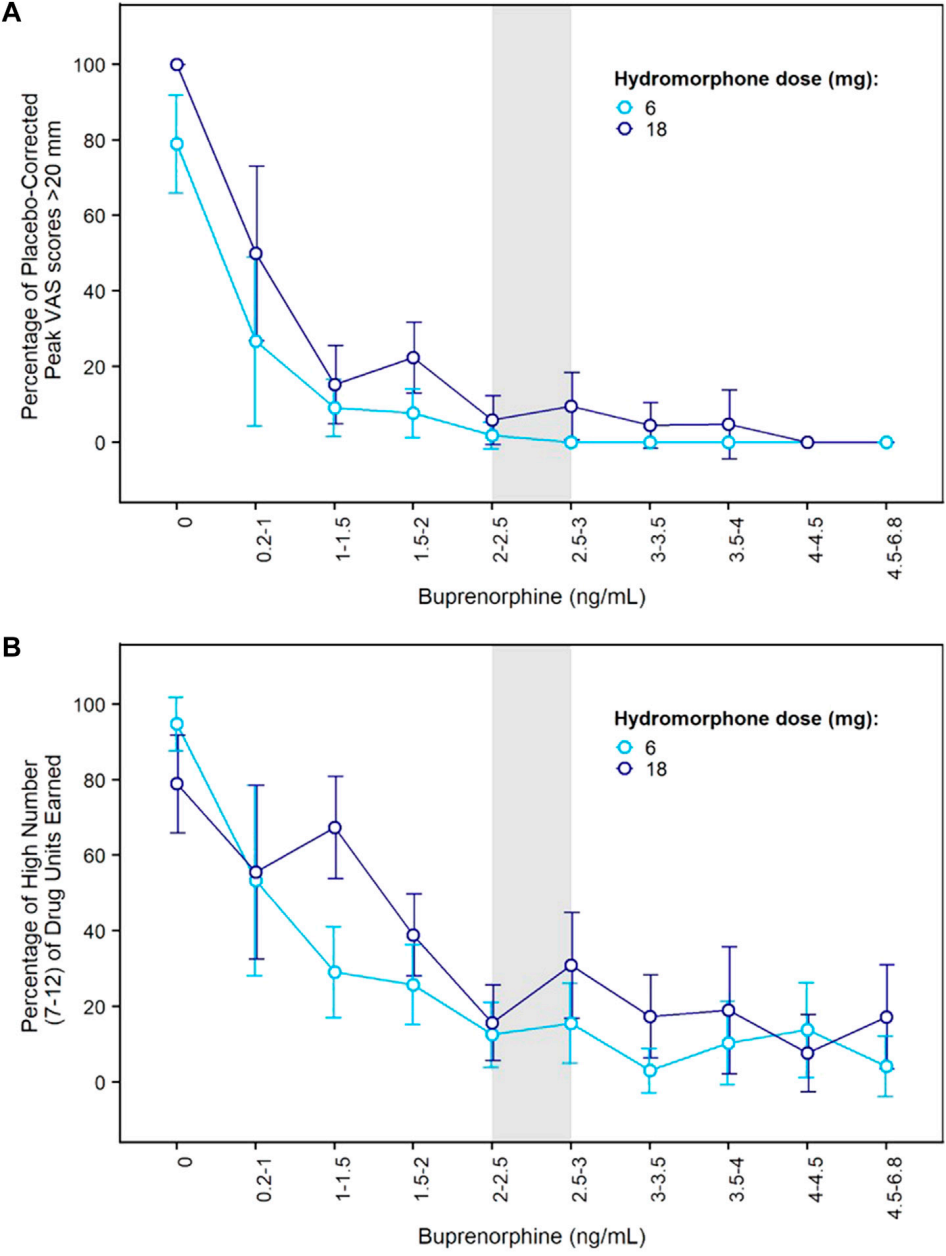
Concentration-Response Relationships for Hydromorphone Subjective and Reinforcing Effects in the Phase 2 Study. **(A)** Percentage of elevated drug liking (defined as placebo-corrected peak VAS score >20 mm) vs. buprenorphine plasma concentration after morning challenge with hydromorphone 6 mg or 18 mg; **(B)** Percentage of high number (7–12) of hydromorphone units earned during afternoon choice sessions vs. buprenorphine plasma concentration after morning challenge with hydromorphone 6 mg or 18 mg. Error bars delineate 95% confidence intervals. VAS: visual analogue scale.

Concentration-response curves indicated a clear effect of buprenorphine plasma concentration on drug liking (Figure 3A). At concentrations ≥ 2 ng/ml, buprenorphine reduced drug liking in most instances, with less than 10% of placebo-corrected scores above 20 mm on the 100-mm drug liking VAS. Additionally, higher buprenorphine plasma concentrations were required to block the effects of 18 mg hydromorphone compared to 6 mg. Due to high within-subject variability in drug liking scores, no modeling of concentration-response data was conducted.

On each challenge day, participants further completed a multi-choice session to evaluate hydromorphone reinforcing effects. Participants could choose on each of 12 trials between $2.00 (natural reward) or 1/12^th^ unit of the challenge dose they received earlier that day. In the absence of any buprenorphine, participants who received 6 or 18 mg hydromorphone earlier in the day mainly chose drug over money with a mean ± SE of 10.5 ± 0.39 and 9.6 ± 0.59 units for hydromorphone vs. 1.3 ± 0.37 and 2.3 ± 0.58 units for money. The same subjects selected money (9.0 ± 0.54) over hydromorphone (2.4 ± 0.51) after receiving placebo in similar conditions. Frequent drug selection decreased when buprenorphine plasma concentration increased and was low at concentrations ≥ 2 ng/ml (Figure 3B). The number of hydromorphone units earned each day was successfully modeled using a mixed-effects logistic regression model for ordinal data (Table 1). In this model, a maximal effect (E_max_) relationship best described the effect of buprenorphine plasma concentration on drug choices. A higher EC_50_ (concentration yielding 50% of E_max_) was estimated for 18 mg hydromorphone (1.44 ng/ml) compared with 6 mg hydromorphone (0.72 ng/ml), indicating that higher concentrations of buprenorphine were needed to attenuate the reinforcing effects of the higher hydromorphone dose. Comparison of observations vs. model predictions after challenge with hydromorphone or placebo is provided in Supplementary Figure S2 for each category of hydromorphone unit earned. Overall, the model adequately described the observed data over the course of the study.

**TABLE 1:** Concentration-response model for the number of hydromorphone units earned during the evaluation of reinforcing effects in the phase 2 study.

Parameter	Description	Estimate (RSE%)	IIV^#^ (RSE%)
α_1_	Intercept at logit level for N_HYD_=0	−0.273 (127)	1.63 (15)
δ_1_	Delta between α_2_ and α_1_	1.55 (17)	-
δ_2_	Delta between α_3_ and α_2_	1.91 (16)	-
δ_3_	Delta between α_4_ and α_3_	1.46 (18)	-
BASE	HYD effect in the absence of BUP on logit scale		
6 mg HYD		5.19 (12)	55.1 (26)
18 mg HYD		4.80 (13)	
EC_50_	BUP plasma concentration (ng/ml) yielding 50% of maximal effect		
6 mg HYD		0.720 (26)	49.8 (24)
18 mg HYD		1.44 (15)	
γ	Hill coefficient	2.04 (27)	-
E_max_	BUP maximal effect on logit scale	1 fixed	-

# IIV was modeled assuming a normal distribution for α_1_ (SD shown) and log-normal distributions for BASE and EC_50_ parameters (CV% shown). For log-normal distributions, CV% was calculated as $100\times \sqrt{\exp\left(\omega^{2}\right)-1}$ where $\omega^{2}$ is the variance of the related subject-specific random effect.

BUP, buprenorphine; CV, coefficient of variation; HYD, hydromorphone; IIV, interindividual variability; N_HYD_, number of hydromorphone units earned; RSE, relative standard error; SD, standard deviation; α_2_, α_3_, α_4_: intercepts at logit level for N_HYD_=3, 6 and 11, respectively.

### 3.2 Efficacy on clinical measures

Data from 489 participants in the Phase 3 double-blind, placebo-controlled efficacy study were used for concentration-response assessments. Supplementary Figure S3 and Supplementary Table S3 summarize participant disposition and baseline characteristics, respectively. Supplementary Figure S4 shows the mean pharmacokinetic profiles of BUP-XR in the Phase 3 study. The maintenance dose of 100 mg maintained the buprenorphine plasma levels achieved with the two initial monthly doses of 300 mg, while the 300-mg maintenance dose provided higher levels at steady-state (6^th^ injection) with a plateau at 5–6 ng/ml.

**FIGURE 4:**
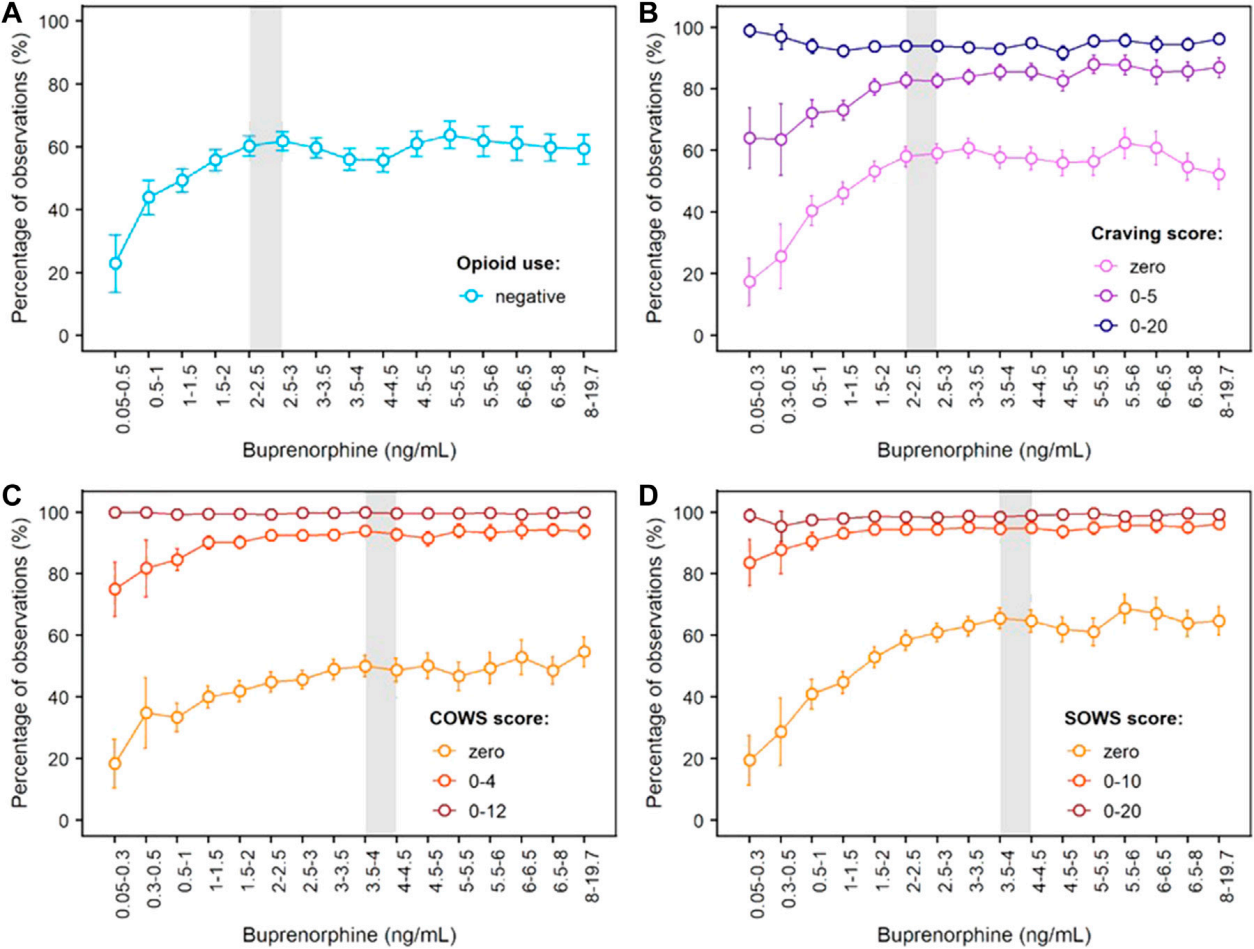
Concentration-Response Relationships for Opioid Use, Opioid Craving and Opioid Withdrawal Symptoms Based on Phase 3 Data. **(A)** Negative opioid use based on urine drug screen and self-report; **(B)** Craving scores measured on the 100-mm Craving visual analogue scale; **(C)** Scores on the Clinical Opiate Withdrawal Scale (COWS); **(D)** Scores on the Subjective Opiate Withdrawal Scale (SOWS). The grey shaded area delineates buprenorphine plasma levels needed to reach the plateau for maximal effect, i.e., 2–3 ng/ml for negative opioid use and opioid craving, and around 4 ng/ml for withdrawal symptoms measured by the clinician (COWS) or the patient (SOWS). Error bars delineate 95% confidence intervals. Exposure-response relationships were assessed from 8,860 (opioid use), 8,345 (craving), 8,850 (COWS) and 8,764 (SOWS) paired concentration/efficacy data.

Concentration-response curves for opioid use (based on urine drug screens and self-reports), craving (measured with the Opioid Craving VAS) and withdrawal symptoms (measured with the COWS and SOWS) are shown in Figure 4. For each measure, buprenorphine efficacy increased with buprenorphine plasma concentration until a plateau for maximal effect was reached. This plateau was achieved at buprenorphine plasma concentrations of 2–3 ng/ml for opioid use and craving, with approximately 60% of observations negative for opioid use, 58% of observations with a craving score of zero, and 85% of observations with a craving score ≤ 5. For opioid withdrawal symptoms, the plateau for maximal response was reached at approximately 4 ng/ml buprenorphine, with larger proportions of zero scores reported on the COWS (50%) and SOWS (65%). However, most participants had COWS scores ≤ 12 or SOWS scores ≤ 10 corresponding to no or mild withdrawal at lower plasma levels, indicating that concentrations of 4 ng/ml may not be necessary to clinically control withdrawal symptoms.

An integrated model for opioid abstinence, craving and dropout was successfully developed from the Phase 3 study data. Mixed-effects logistic regression models for binary and ordinal data accurately described longitudinal measures of opioid use and opioid craving (categorized as 0, 1–5, 6–20 and >20). In both models, the effect of buprenorphine plasma concentration was best characterized by an E_max_ relationship in agreement with the observed concentration-response curves. Model parameter estimates are displayed in Table 2 (opioid abstinence) and Table 3 (craving); equations are provided in the supplemental material.

**TABLE 2:** Concentration-response model for opioid abstinence in the phase 3 study.

Parameter	Description	Estimate (RSE%)	IIV (RSE%)^#^
α	Intercept at logit level (300/300 mg and PBO)	−3.30 (16)	2.64 (6.0)
E_max_	Maximal BUP effect	4.86 (9.7)	38.7 (32)
EC_50_	BUP concentration yielding 50% of E_max_	1.21 (44)	151 (0.39)
β_α_ (300/100)	Relative intercept for 300/100 mg compared to 300/300 mg and PBO	0.794 (10)	−
β_α_ (*OPRD1* TC)	Fractional change in α for *OPRD1* TC genotype (rs678849)	0.133 (150)	−
β_α_ (*OPRD1* TT)	Fractional change in α for *OPRD1* TT genotype (rs678849)	0.309 (92)	−
β_EC50_ (INJUSE)	Fractional increase in EC_50_ for use of opioids by injectable route at baseline	2.57 (47)	−
β_EC50_ (*OPRD1* TC)	Fractional decrease in EC_50_ for *OPRD1* TC genotype (rs678849)	−0.713 (19)	−
β_EC50_ (*OPRD1* TT)	Fractional decrease in EC_50_ for *OPRD1* TT genotype (rs678849)	−0.937 (4.0)	−
β_EC50_ (RACE)	Fractional decrease in EC_50_ for African Americans	−0.113 (910)	−
β_Emax_ (EMPLY)	Fractional increase in E_max_ for employed participants at baseline	0.427 (37)	−
β_Emax_ (RACE)	Fractional decrease in E_max_ for African Americans	−0.311 (31)	−

# IIV was modeled assuming a normal distribution for α (SD shown) and log-normal distributions for E_max_ and EC_50_ (CV% shown). For log-normal distributions, CV% was calculated as $100\times \sqrt{\exp\left(\omega^{2}\right)-1}$ where $\omega^{2}$ was the variance of the related subject-specific random effect.

BUP, buprenorphine; CV, coefficient of variation; IIV, interindividual variability; PBO, placebo; RSE, relative standard error; SD, standard deviation.

**TABLE 3:** Concentration-response model for opioid craving in the phase 3 study.

Parameter	Description	Estimate (RSE%)	IIV (RSE%)^#^
α_1_	Intercept at logit level for zero craving (300/300 mg and PBO)	−2.41 (6.8)	2.10 (4.7)
α_1_ (300/100)	Intercept at logit level for zero craving (300/100 mg)	−1.87 (11)	−
δ_1_	Delta between α_2_ and α_1_	2.28 (1.7)	−
δ_2_	Delta between α_3_ and α_2_	1.85 (2.5)	−
E_max_	Maximal BUP effect	2.87 (7.2)	101 (7.0)
EC_50_	BUP concentration yielding 50% of E_max_	2.45 (15)	−
β_Emax_ (BMI)	Coefficient for BMI (power model) on E_max_	0.853 (37)	−

#IIV was modeled assuming a normal distribution for α_1_ (SD shown) and a log-normal distribution for E_max_ (CV% shown). For the log-normal distribution, CV% was calculated as $100\times \sqrt{\exp\left(\omega^{2}\right)-1}$ where $\omega^{2}$ was the variance of the related subject-specific random effect.

BMI, body mass index; BUP, buprenorphine; CV, coefficient of variation; IIV, interindividual variability; PBO, placebo; RSE, relative standard error; SD, standard deviation.

Several statistically significant covariates were retained in the opioid abstinence model after forward/backward selection. Participants who injected opioids at baseline had a 3.6-fold higher buprenorphine EC_50_ (4.3 ng/ml) compared to those who used opioids by non-injectable routes (1.2 ng/ml). Hence, injecting opioid users seemed to require higher buprenorphine concentrations to maximize opioid abstinence. Genetic variant rs678849 on the *delta* opioid receptor gene *OPRD1* was also a significant covariate on the EC_50_, with a 94% and 71% lower EC_50_ in participants with TT or TC genotype, respectively, compared to participants with the CC genotype. Self-identified Black/African Americans (hereafter African Americans) showed an 11% lower buprenorphine EC_50_, but uncertainty in the estimate was large. Regarding buprenorphine maximal effect E_max_, it was significantly influenced by race (-31% in African Americans) and employment (+43% for participants employed at baseline). A small difference in the baseline probability of opioid abstinence (prior to any buprenorphine treatment) was found between BUP-XR lower dose group (6.8%) and other treatment groups (placebo and BUP-XR higher dose; 3.6%), which was attributed to chance. For the opioid craving model, body mass index was the only statistically significant covariate retained after forward/backward selection with an effect on E_max_. This covariate, however, only explained 1% of the variability and was not considered clinically relevant.

Given substantial dropout in the Phase 3 study, a model for dropout was developed in parallel with the models for opioid abstinence and opioid craving under the missing-at-random assumption. Dropout was successfully predicted from baseline participant characteristics and observed measures of efficacy (craving) using a Gompertz hazard model. Equations are provided in the supplemental material with model parameter estimates displayed in Table 4. Because dropout rates were similar between the two BUP-XR maintenance dosing regimens (100 mg or 300 mg), treatment (active vs. placebo) was used as a covariate in place of buprenorphine plasma concentration. Dropout rate was estimated to be two times lower for active treatment compared to placebo in line with observations (observed dropout rates were 33–34% for BUP-XR groups vs. 64% for placebo). Results of the covariate analysis indicated that opioid craving (included as a time-varying covariate) was a major predictor of dropout whereas opioid use was not significant. Compared to craving scores ≤ 5, craving scores > 20 were associated with a 3.0- to 3.6-fold increase in dropout rate across treatment groups whether participants received BUP-XR or placebo. Other significant predictors of dropout were race, age, and disease severity at baseline assessed with the Clinical Global Impression-Severity scale. In all treatment groups, baseline hazard (β_0_) was reduced by approximately 40% in African Americans compared to other participants (mainly white). Interestingly, age only had an effect in the placebo group, with the highest dropout observed in participants below 30 years of age and lowest in participants above 50 years of age. Age was not a significant predictor in BUP-XR treatment groups, suggesting that once under active treatment, age did not affect treatment retention. Finally, placebo-treated participants with a Clinical Global Impression-Severity score ≥ 4 (moderately to severely ill patients) showed better retention in the trial.

**TABLE 4:** Dropout model based on phase 3 data.

Parameter	Description	Estimate (RSE%)
β_0, TRT_	Baseline hazard constant in BUP-XR groups	0.00459 (17)
β_0, PBO_	Baseline hazard constant in PBO group	0.0102 (35)
β_1, TRT 2_	Coefficient for craving = 1–5 (category 2) in BUP-XR groups	0.869 (23)
β_1, TRT 3_	Coefficient for craving = 6–20 (category 3) in BUP-XR groups	1.29 (28)
β_1, TRT 4_	Coefficient for craving > 20 (category 4) in BUP-XR groups	2.60 (26)
β_1, PBO 2_	Coefficient for craving = 1–5 (category 2) in PBO group	0.472 (37)
β_1, PBO 3_	Coefficient for craving = 6–20 (category 3) in PBO group	0.534 (43)
β_1, PBO 4_	Coefficient for craving > 20 (category 4) in PBO group	1.72 (32)
β_2_	Coefficient for race (African Americans) on baseline hazard (common to PBO and BUP-XR groups)	0.602 (19)
β_3, PBO_	Coefficient for age (power model) on baseline hazard in PBO group only	−1.64 (27)
β_4, PBO_	Coefficient for CGI-S ≤ 3 on baseline hazard in PBO group only	3.86 (27)
ke _TRT_	Rate constant for exponential decrease in hazard over time (Gompertz model) in BUP-XR groups	0.00763 (22)
ke _PBO_	Rate constant for exponential decrease in hazard over time (Gompertz model) in PBO group	0.00895 (37)

CGI-S, Clinical Global Impression-Severity scale; PBO, placebo; RSE, relative standard error.

Joint predictions from the dropout, opioid use and opioid craving models adequately described changes over time in the number of participants negative and positive for opioid use in the Phase 3 trial, as well as the number of participants within each level of opioid craving (Supplementary Figure S5). Kaplan Meier plots (Supplementary Figures S6–S9) show close agreement between predicted and observed dropout rates within each treatment group and illustrate the effect of baseline subject characteristics on dropout rates.

## 4 Discussion

The objective of our analyses was to expand previous quantitative work from small but seminal laboratory studies that established correlations between buprenorphine plasma concentrations, brain MOR occupancy, and pharmacodynamic effects (Kuhlman et al., 1998; Greenwald et al., 2003; Comer et al., 2005; Greenwald et al., 2007; Greenwald et al., 2014). Our results show clear concentration-response relationships across efficacy measures. For opioid abstinence and opioid craving (assessed in the pivotal Phase 3 study), a plateau for maximal efficacy was observed in the overall sample of patients at buprenorphine plasma concentrations of 2–3 ng/ml (corresponding to 68–75% MOR occupancy based on Nasser et al., 2014). Buprenorphine effects on opioid withdrawal symptoms were maximized at plasma concentrations of 4 ng/ml (estimated 78% MOR occupancy), with greater proportions of zero scores reported on the COWS (clinician’s evaluation) and SOWS (participant’s self-evaluation). We note, however, that many participants reported scores of no to mild withdrawal at relatively low buprenorphine concentrations. Although observations at these low concentrations may be confounded by uncontrolled opioid use, they suggest that 4 ng/ml buprenorphine may not be necessary to reduce withdrawal symptoms below clinically significant levels. This aligns with previous results from laboratory studies which required opioid abstinence prior to pharmacodynamic evaluations and showed that opioid withdrawal symptoms could be clinically controlled at buprenorphine plasma concentrations of 1 ng/ml (estimated 55% MOR occupancy) or higher (Kuhlman et al., 1998; Greenwald et al., 2007; Greenwald et al., 2014).

In the Phase 2 study, opioid blockade was assessed after two monthly injections of BUP-XR (300 mg) which delivered sustained buprenorphine plasma concentrations of 2–3 ng/ml on average. Non-inferiority analysis of hydromorphone vs. placebo “drug liking” effects demonstrated that participants exposed to these concentrations did not value hydromorphone over placebo. This finding was further supported by concentration-response analyses, indicating that 2–3 ng/ml buprenorphine blocked hydromorphone liking in most study participants as well as reinforcing effects of hydromorphone with markedly reduced selection of hydromorphone over money.

Previous blockade studies (listed in Supplementary Table S5) have reported opioid blockade at various buprenorphine doses depending on buprenorphine formulation and route of administration (subcutaneous injection: 8 mg, transmucosal solution: 8–16 mg; transmucosal tablet: 8–32 mg). Heterogeneity in the results can be explained by differences in buprenorphine bioavailability (subcutaneous injection > transmucosal solution > transmucosal tablet; see Chiang and Hawks, 2003), opioid agonist selection (hydromorphone, morphine, heroin), opioid agonist dose (larger doses likely require higher buprenorphine concentrations for blockade), and other differences in study design such as the time of opioid challenge after buprenorphine dose. It is noteworthy that most studies included a small number of subjects and that some studies reported large variability in individual response. In terms of buprenorphine exposure, transmucosal tablets of 8 to 24 mg/day deliver average plasma concentrations of 1.4 to 2.8 ng/ml, respectively, but there are wide variations over time from peak (4.3–8.9 ng/ml) to trough (0.7–1.4 ng/mL)^3^. In our Phase 2 study, opioid challenges were performed at the end of each week when buprenorphine plasma concentrations were relatively stable (“plateau” phase; see Figure 2). One previous opioid blockade study used an extended-release weekly formulation of buprenorphine and measured buprenorphine plasma concentrations at the time of opioid challenges (6 mg and 18 mg hydromorphone) (Walsh et al., 2017). Mean buprenorphine plasma concentrations associated with opioid blockade were in the 2–4 ng/ml range (1.9–3.8 ng/ml) with one value at 1.5 ng/ml. Although that study used a weekly rather than monthly formulation, its findings are not inconsistent with our concentration-response curves which show efficacy at plasma levels of 1–1.5 and 1.5–2 ng/ml with additional improvement at 2–3 ng/ml. Differences in efficacy between concentrations of 1–1.5 ng/ml and 2–3 ng/ml were more evident on hydromorphone reinforcing effects (Figure 3B). It is noteworthy that 18 mg hydromorphone blockade was not achieved in our Phase 2 study when the plasma concentration averaged 1.2 ng/ml during transmucosal buprenorphine run-in.

Effective opioid blockade is explained by the binding characteristics of buprenorphine at MORs. Buprenorphine has high affinity for MORs and slow receptor association and dissociation compared to other opioids (Boas and Villiger, 1985; Volpe et al., 2011). Competitive binding studies of buprenorphine and fentanyl (potent MOR full agonist) showed that buprenorphine displaced fentanyl in a concentration-dependent manner (Boas and Villiger, 1985). However, buprenorphine was displaced with only very high concentrations of other opioids, which explains the difficulty of reversing buprenorphine effects by naloxone (Yassen et al., 2007a). When buprenorphine preceded fentanyl administration, buprenorphine suppressed almost completely any subsequent binding of fentanyl at equimolar concentrations (Boas and Villiger, 1985). These *in vitro* findings fully align with results of a recent clinical study in opioid-tolerant subjects where sustained exposure to buprenorphine reduced the frequency and magnitude of respiratory depression induced by fentanyl (Moss et al., 2022; Olofsen et al., 2022). In that study, buprenorphine’s blockade was concentration-dependent, with plasma concentrations of 2 and 6 ng/ml achieving greater suppression of fentanyl respiratory effects compared to 1 ng/ml concentration. Although these findings show that buprenorphine is able to block fentanyl-induced respiratory depression, no buprenorphine study assessing the blockade of fentanyl subjective and reinforcing effects has been conducted to the best of our knowledge.

The extent of opioid blockade obviously depends on the relative affinity and concentration (dose) of other opioids. In our Phase 2 study, slightly higher buprenorphine concentrations were needed to block the effects of 18 mg hydromorphone compared to 6 mg hydromorphone. Similar effect of the agonist challenge dose was observed in previous studies (Bickel et al., 1988; Rosen et al., 1994; Walsh et al., 1995; Schuh et al., 1999; Comer et al., 2001; Comer et al., 2005). Additionally, our modeling results suggest that subjects injecting opioids (thereby achieving higher opioid concentrations at MORs) would require higher buprenorphine levels to achieve optimal reductions in their opioid use. This finding was supported by *post hoc* analyses of the Phase 3 study showing greater abstinence rates among injecting opioid users during BUP-XR 300 mg maintenance dosing (average concentration: 6.5 ± 2.1 ng/ml) compared to BUP-XR 100 mg maintenance dosing (average concentration of 3.2 ± 0.8 ng/ml) while such difference was not observed in non-injecting opioid users despite comparable pharmacokinetic profiles (Fox et al., 2019). Nonetheless, there was no overall difference in efficacy between the two BUP-XR maintenance dosing regimens (Haight et al., 2019), indicating that concentrations of 2–3 ng/ml provided by the 100-mg maintenance dose were likely sufficient in most study participants. No effect of opioid injection status on hydromorphone choices was observed in the Phase 2 study but the sample size was limited.

A potential limitation of our work is that BUP-XR rapidly provided buprenorphine plasma concentrations in the range of 2–3 ng/ml so that the lower part of the concentration-response curves was mainly informed by buprenorphine concentrations measured early in treatment (mainly after the first BUP-XR injection). Still, concentration-response relationships were well characterized (small error bars) and no relevant time effect was evidenced. Notably, exposure-response curves were superimposable (with similar E_max_) when stratified by dosing interval or dosing regimen despite very different plasma concentrations over time (data not shown). Another potential limitation is the delay (hysteresis) between buprenorphine plasma concentration and brain MOR occupancy. Previous modeling estimated a half-life of 75 min for buprenorphine distribution to the biophase and a half-life of 68 min for buprenorphine dissociation from MORs (Yassen et al., 2007b). Since measures of efficacy in the Phase 3 study were performed weekly with one measure at the peak concentration for each BUP-XR injection and at the trough concentration for transmucosal buprenorphine at the end of run-in, it was not possible to model delays of this magnitude. At the time of the weekly visits (representing most observations), buprenorphine plasma concentrations were in the “plateau” phase and the equilibrium between plasma and biophase concentrations was reasonably assumed. No change in the shape of the concentration-response curves was noted after excluding BUP-XR peak concentrations or transmucosal buprenorphine trough concentrations from the analysis (data not shown).

The opioid abstinence model provided additional insights on factors modulating treatment response. Notably, our analysis revealed a significant effect of rs678849 variant in *OPRD1* encoding the *delta*-opioid receptor, with a 94% and 71% lower EC_50_ in participants with TT or TC genotypes, respectively, compared to participants with CC genotype. Earlier studies identified this variant as a predictor of buprenorphine treatment response, but those effects were only seen in African Americans, not European Americans (Crist et al., 2013; Crist et al., 2019). Here, 130 African Americans and 349 European Americans were included in the Phase 3 study and, although rs678849 effect was estimated from the totality of the data, the effect remained significant when the analysis was stratified by race. Of note, analysis of the same Phase 3 data using more conventional methodology showed a moderating effect of rs678849 on buprenorphine treatment response in European Caucasian but not African-descent groups (Kranzler et al., 2021). Given that MORs are the classical target of buprenorphine, the mechanisms by which *OPRD1* rs678849 may regulate treatment response are unclear as discussed by Kranzler et al. (2021). It is noteworthy that buprenorphine binds with high affinity to the *delta*-opioid receptor and produces antagonist effects albeit at a lower potency compared to *mu* or *kappa* receptor-mediated effects (Negus et al., 2002). Overall, genetic studies for OUD have provided mixed results, most likely because single genetic variants have relatively small effect sizes (Agrawal et al., 2012; Zhou et al., 2020), and further studies are needed to confirm the present findings.

Both route of opioid use and genetic variation in *OPRD1* (rs678849) were found to affect buprenorphine EC_50_, meaning that these factors can potentially influence the selection of buprenorphine dose for a given patient. In contrast, employment and race affected E_max_ which is buprenorphine maximal efficacy reached at infinite buprenorphine plasma concentration: E_max_ increased by 43% when participants were employed at baseline and decreased by 31% in African Americans. While not directly demonstrated for buprenorphine, those effects are consistent with findings reported for other OUD medications (e.g., methadone) although mixed results have been generated, especially for self-identified race (Black vs. White) (Marsch et al., 2005; Dreifuss, et al., 2013). It is possible that the effect of race is confounded by other sociodemographic differences. Employment was previously cited as a protective factor in providing “alternative, non-drug related sources of reinforcement” (Marsch et al., 2005). No significant gender effect was evidenced in the present analysis. Conflicting results on gender have been reported in the literature; in some analyses, male gender was associated with poorer buprenorphine treatment outcome (Marsch et al., 2005; Dreifuss, et al., 2013).

Finally, retention in treatment was successfully predicted from baseline participant characteristics and recorded measures of efficacy using a Gompertz hazard model. Previous clinical trials showed treatment retention increased with transmucosal buprenorphine dose, with 60% retention at the highest dose of 30–32 mg/day (Ling et al., 1998; Hser et al., 2014). Similarly, a meta-analysis of 21 controlled, double-blind clinical studies indicated better retention at transmucosal buprenorphine doses of 16–32 mg/day compared to lower doses (Fareed et al., 2012). In the present Phase 3 study, similar retention was observed between the two dosing regimens of BUP-XR, with values of 66–67% consistent with maximal retention rates previously reported for transmucosal buprenorphine. Notably, monthly doses of 100 mg and 300 mg BUP-XR provided average plasma concentrations equivalent to 24 mg transmucosal buprenorphine or above^4^. A twice-higher dropout rate was estimated for the placebo group. The covariate analysis indicated that opioid craving, but not opioid use, was an important predictor of dropout across all treatment groups including placebo. Craving scores > 20 were associated with up to 3.6-fold higher dropout rates compared to craving scores ≤ 5. Altogether, these results emphasize the need to monitor patient’s craving in clinical practice and foster the development of validated measurement tools. Race was also identified as a significant predictor of treatment retention, with 40% lower dropout rates in African Americans whether those participants received BUP-XR or placebo. Underlying reasons for this finding are unclear and may be confounded with other factors. Age was previously reported as an important predictor of dropout in several buprenorphine studies, with younger age associated with shorter retention (Hser et al., 2014; Schuman-Olivier et al., 2014; Marcovitz et al., 2016). A similar age effect was seen in the placebo group, but not in BUP-XR treatment groups, suggesting that once on BUP-XR age had no impact on treatment retention.

Overall, our data provide key information on buprenorphine plasma levels needed to optimize treatment outcomes in patients with moderate to severe OUD. Achieving opioid blockade is critical to reduce abuse-related subjective ratings (e.g., “drug liking”) and reinforcing (seeking/self-administration) effects of opioids and should be the primary criterion guiding selection of buprenorphine maintenance dose (Greenwald et al., 2014). Craving is also an important component of the disease as it reflects memories of drug or drug-associated environmental cues and can lead to opioid use even if opioid blockade is achieved (Ling et al., 2019a). Here we show that 2–3 ng/ml buprenorphine provided consistent opioid blockade in the Phase 2 study and optimized control of opioid craving and withdrawal symptoms. Taken together, these effects translated in the Phase 3 trial into reductions in opioid use, with the same plasma levels producing effects at the plateau of the concentration-response curve. Although injecting opioid users appeared to benefit from higher concentrations delivered by BUP-XR 300-mg maintenance dose, buprenorphine plasma levels of 2–3 ng/ml achieved with BUP-XR 100-mg maintenance dose were sufficient in the majority of participants. These concentration-response findings also translated into broader clinical benefits. Evaluation of participant-centered outcomes in Phase 3 studies revealed high medication satisfaction (88–89%) after BUP-XR treatment as well as meaningful life changes with increased employment, decreased healthcare utilization, improved treatment effectiveness and reduction in addiction severity (Ling et al., 2019b; Ling et al., 2020a). Also, the longer the duration of BUP-XR treatment, the better the outcomes 1 year after treatment cessation, with a higher likelihood of sustained opioid abstinence after 12 months of treatment compared to ≤ 2 months of treatment (75.3% vs. 24.1%; *p* = 0.001) (Ling et al., 2020b). Similarly, improvements in employment, quality of life and treatment satisfaction were observed in a recent 12-month prospective open-label study of BUP-XR in Australia (Farrell et al., 2022). In that study, the odds of use of all illicit substances except cannabis decreased significantly with time retained in treatment, together with a significant reduction in the odds of moderate-severe depression and a significant decline in pain. No concentration-response analyses were performed on participant-centered outcomes and further research might provide additional insights.

Last but not least, BUP-XR was well tolerated with a safety profile similar to transmucosal buprenorphine, except for injection site reactions, which were mostly mild to moderate and not treatment limiting (Haight et al., 2019; Andorn et al., 2020). In the Phase 3 study, treatment-emergent adverse events (TEAEs) were reported in a higher percentage for BUP-XR dosing regimens (300/300 mg: 67% and 300/100 mg: 76%) compared to placebo (56%); the most common TEAEs were headache (8% and 9% vs. 6%), constipation (8% and 9% vs. 0%), nausea (8% and 9% vs. 5%) and injection-site pruritis (9% and 6% vs. 4%) (Haight et al., 2019). Serious TEAEs were reported in 3%, 2% and 5% of participants in the 300/300 mg, 300/100 mg and placebo groups, respectively. An open-label long-term safety study of BUP-XR demonstrated similar acceptable safety profile over 12 months with a lower incidence of TEAEs in the second 6 months of treatment compared to the first 6 months of treatment (Andorn et al., 2020).

In conclusion, our findings add to growing evidence that patients with OUD significantly benefit from sufficient buprenorphine exposure to achieve treatment success. Importantly, we provide information on buprenorphine plasma levels that optimized treatment outcomes in the majority of patients, therefore increasing the likelihood of success when treating patients individually. We also point towards the benefit of extended-release buprenorphine formulations that can sustain these levels throughout a longer dosing period.

## Data Availability

Data are available from the authors upon reasonable request. All requests for raw and analysed data will be promptly reviewed by the sponsor delegate to verify if the request is subject to any confidentiality obligations. Patient-related data not included in the paper were generated as part of clinical trials and may be subject to patient confidentiality. Any data that can be shared will be released via a data use agreement.

## Footnotes

1
https://www.cdc.gov/nchs/nvss/vsrr/drug-overdose-data.htm.

2 Sublocade prescribing information. Indivior, Inc., North Chesterfield, VA. June 2021. https://www.accessdata.fda.gov/drugsatfda_docs/label/2022/209819s010lbl.pdf.

3 Sublocade prescribing information. Indivior, Inc., North Chesterfield, VA. June 2021. https://www.accessdata.fda.gov/drugsatfda_docs/label/2022/209819s010lbl.pdf.

4 Sublocade prescribing information. Indivior, Inc., North Chesterfield, VA. June 2021. https://www.accessdata.fda.gov/drugsatfda_docs/label/2022/209819s010lbl.pdf.
