# Supplementary material for: Buprenorphine exposure levels to optimize treatment outcomes in opioid use disorder
Source: Front Pharmacol. 2022 Nov 18;13:1052113. doi: 10.3389/fphar.2022.1052113 (PMC9715596; doi:10.3389/fphar.2022.1052113)
Supplement: Supplementary file 1 [file DataSheet1.docx]

**Supplemental Material**

**Table S1. Number of Computer Clicks Required to Complete Each Trial During Afternoon Choice Sessions Assessing Hydromorphone Reinforcing Effects in the Phase 2 Study**

| **Highest Level of Drug Units Earned** | **Number of Computer Clicks** |
| --- | --- |
| 1 | 5 |
| 2 | 40 |
| 3 | 70 |
| 4 | 120 |
| 5 | 180 |
| 6 | 260 |
| 7 | 395 |
| 8 | 555 |
| 9 | 775 |
| 10 | 1110 |
| 11 | 1558 |
| 12 | 2160 |

Hydromorphone reinforcing effects were assessed on hydromorphone/placebo challenge days. Those procedures occurred at least 5 hours after the morning challenge and consisted of a series of 12 trials. On each trial, the participant could choose to earn $2.00 (natural reward) or 1/12^th^ unit of the morning dose of hydromorphone or placebo. For each trial, the participant had to ‘mouse’-click repeatedly on a “drug” or “money” icon on a computer screen. Across the 12 trials within the session, the computer “mouse”-clicking response requirement on each option increased according to a progressive ratio schedule of reinforcement**.** To receive all the drug or money, participants were required to click 7228 times.

**Table S2. Urine Drug Screens in the Phase 3 Study**

**Urine Drug Screen Immunoassays**

| **Drug/Drug Class** | **Cut-off (ng/mL)** | **Calibrator** |
| --- | --- | --- |
| Amphetamine | 1000 | d-Methamphetamine |
| Barbiturates | 300 | Secobarbital |
| Benzodiazepines | 300 | Lormetazepam |
| Buprenorphine | 5 | Buprenorphine |
| Cannabinoids | 50 | 11-nor-delta9-THC-9-COOH |
| Cocaine Metabolite | 300 | Benzoylecgonine |
| Methadone | 300 | Methadone |
| Opiates | 300 | Morphine |
| Oxycodone | 300 | Oxycodone |
| Phencyclidine | 25 | Phencyclidine |
| Source: ACM Global Central Laboratory | | |

**Urine Drug Screen** **Confirmatory Testing Information**

| **Methodology** | **Confirmation Component** | **Cut-off (ng/mL)** | **Calibrator (ng/mL)** | **Hydrolysis (Yes or No)** | **Limit of Detection**  **(ng/mL)** | **Limit of Quantitation (ng/mL)** | **Limit of Linearity (ng/mL)** |
| --- | --- | --- | --- | --- | --- | --- | --- |
| GC/MS | Methadone | 300 | 300 | No | 100 | 100 | 3000 |
|  |  |  |  |  |  |  |  |
| GC/MS | Codeine | 50 | 300 | Yes | 50 | 100 | 4000 |
|  | Morphine | 50 | 300 | Yes | 50 | 100 | 2000 |
|  | Hydrocodone | 50 | 300 | Yes | 50 | 100 | 3000 |
|  | Hydromorphone | 50 | 300 | Yes | 50 | 100 | 2000 |
|  | Oxycodone | 50 | 300 | Yes | 50 | 100 | 3000 |
|  | Oxymorphone | 50 | 300 | Yes | 50 | 100 | 2000 |
| GC/MS: Gas chromatography/mass spectrometry  Note: Hydrolysis is a process used to treat samples prior to analysis by GC/MS. Many drugs, such as opiates, benzodiazepines and buprenorphine are metabolised in the body. Part of this metabolism is the conjugation of the drug molecule to a glucuronic acid molecule. The glucuronide conjugated drug is more water soluble and more easily excreted in urine. Prior to GC/MS analysis, this glucuronide bond must be cleaved via hydrolysis.  Source: ACM Global Central Laboratory | | | | | | | |

Table S3. Baseline Characteristics of Participants from Phase 2 and Phase 3 Studies of BUP-XR Included in Concentration-Responses Analyses

| Participant Characteristics | Phase 2 | Phase 3 | | |
| --- | --- | --- | --- | --- |
|  |  | 300/100 mg | 300/300 mg | Placebo |
| Number | 39 | 194^#^ | 196^#^ | 99^#^ |
| Age (years) | 34.6 (8.9) | 40.4 (11.2) | 39.3 (11.0) | 39.2 (11.0) |
| Weight (kg) | 79.6 (11.2) | 76.2 (15.9) | 79.3 (16.1) | 75.1 (16.0) |
| BMI (kg/m^2^) | 25.4 (3.0) | 25.2 (4.2) | 26.2 (4.4) | 25.2 (4.3) |
| Sex  Male  Female | 35 (89.7)  4 (10.3) | 128 (66.0)  66 (34.0) | 132 (67.3)  64 (32.7) | 64 (64.6)  35 (35.4) |
| Race  White  Black/African American  Other | 25 (64.1)  12 (30.8)  2 (5.1) | 132 (68.0)  56 (28.9)  6 (3.1) | 140 (71.4)  54 (27.6)  2 (1.0) | 77 (77.8)  20 (20.2)  2 (2.0) |
| Use of Injectable Opioids  No  Yes | 10 (25.6)  29 (74.4) | 110 (56.7)  84 (43.3) | 116 (59.2)  80 (40.8) | 49 (49.5)  50 (50.5) |
| CGI-S status  Normal  Borderline  Mildly ill  Moderately ill  Markedly ill  Severely ill  Missing | NA | 19 (9.8)  2 (1.0)  8 (4.1)  72 (37.1)  67 (34.5)  15 (7.7)  11 (5.7) | 33 (16.8)  2 (1.0)  14 (7.1)  63 (32.1)  63 (32.1)  11 (5.6)  10 (5.1) | 7 (7.1)  2 (2.0)  6 (6.1)  37 (37.4)  33 (33.3)  2 (2.0)  12 (12.1) |
| Employed  No  Yes  Missing | NA | 130 (67.0)  55 (28.4)  9 (4.6) | 113 (57.7)  76 (38.8)  7 (3.6) | 55 (55.6)  34 (34.3)  10 (10.1) |
| Health Insurance  No  Yes  Missing | NA | 77 (39.7)  108 (55.7)  9 (4.6) | 78 (39.8)  111 (56.6)  7 (3.6) | 37 (37.4)  52 (52.5)  10 (10.1) |
| OPRD1 (rs678849)  CC  TC  TT  Missing | NA | 66 (34.0)  80 (41.2)  38 (19.6)  10 (5.2) | 68 (34.7)  78 (39.8)  37 (18.9)  13 (6.6) | 33 (33.3)  47 (47.5)  15 (15.2)  4 (4.0) |
| BMI: body mass index; CGI-S: Clinical Global Impression-Severity scale; NA: not available; OPRD1: gene encoding for the *delta*-opioid receptor  Results are shown as mean (SD) for continuous variables and counts (percentage) for categorical variables.  ^#^ One site was closed by the sponsor because of compliance issues and all 15 participants from that site were excluded from efficacy analyses. Hence, from the 504 participants enrolled, 489 were included in the concentration-response analyses. | | | | |

Table S4. Medians of Placebo-corrected Peak Drug Liking VAS Scores by Hydromorphone Dose Per Week in the Phase 2 Study

|  |  | Median (95% CI) Placebo-Corrected  Peak Drug Liking VAS Score | |
| --- | --- | --- | --- |
| Week | N^1^ | 6 mg hydromorphone | 18 mg hydromorphone |
| Screening | 38 | 59.5 (40.0, 66.0) | 72.0 (63.0, 80.0) |
| 0^2^ | 38 | 1.0 (0.0, 6.0) | 11.0 (4.0, 27.0) |
| 1 | 38 | 0.5 (0.0, 3.0) | 2.0 (0.0, 10.0) |
| 2 | 34 | 0.0 (0.0, 1.0) | 1.5 (0.0, 8.0) |
| 3 | 33 | 0.0 (0.0, 1.0) | 0.0 (0.0, 6.0) |
| 4 | 30 | 1.0 (0.0, 2.0) | 1.5 (0.0, 7.0) |
| 5 | 28 | 0.0 (0.0, 0.0) | 0.0 (0.0, 0.0) |
| 6 | 27 | 0.0 (0.0, 1.0) | 0.0 (0.0, 1.0) |
| 7 | 26 | 0.0 (0.0, 0.0) | 0.0 (0.0, 1.0) |
| 8 | 24 | 0.0 (-1.0, 1.0) | 0.0 (0.0, 1.0) |
| 9 | 22 | 0.0 (-1.0, 1.0) | 0.0 (-1.0, 0.0) |
| 10 | 18 | 0.0 (0.0, 1.0) | 0.0 (0.0, 1.0) |
| 11 | 18 | 0.0 (0.0, 1.0) | 0.0 (0.0, 4.0) |
| 12 | 17 | 0.0 (0.0, 0.0) | 0.0 (0.0, 5.0) |
| CI: confidence interval; VAS: visual analogue scale  ^1^ Number of subjects having completed challenge results for placebo, 6 mg hydromorphone and 18 mg hydromorphone.  ^2^ End of transmucosal buprenorphine treatment period | | | |

**Table S5. List of Published Opioid Blockade Studies for Buprenorphine^*^**

| **Publication** | **Sample size** | **Opioid Agonist** | **BUP Dose** | **Results** |
| --- | --- | --- | --- | --- |
| Jasinski 1978 | N=5 | Morphine SC  (0, 15, 30, 60, 85, 120 mg) at 1.5 hours or 25.5 hours post BUP dose | SC BUP  0, 8 mg/day | 8 mg of SC BUP blocked the effects of morphine up to 120 mg.  Opioid blockade lasted for at least 29.5 hours when assessed for 30 mg morphine. |
| Mello & Mendelson 1980 | N=10 | Heroin IV  (up to 21 or 40.5 mg/day) | SC BUP  0, 4, 8 mg/day | SC BUP significantly suppressed self-administration of heroin over 10 days. BUP effects were dose-dependent: 8 mg BUP reduced heroin use by 69-98% compared to 45% for 4 mg BUP. |
| Bickel 1988 | N=5 | Hydromorphone SC  (0, 6, 18 mg) at 24 hours post BUP dose | SL BUP (solution)  2, 4, 8, 16 mg/day | Opioid blockade was dose-dependent. Maximal blockade of subjective effects was evident at the highest dose of SL BUP investigated (16 mg). |
| Rosen 1994 | N=6 | Hydromorphone IM  (0, 6, 18 mg) at 24, 48, and 72 hours post BUP dose | SL BUP (solution)  2, 6, 12 mg/day | Data showed a significant blockade of "high" at 12 mg of SL BUP. Opioid blockade persisted across 72 hours after the last BUP dose. |
| Walsh 1995 | N=9 | Hydromorphone IM  (0, 1, 4 mg) at 24 hours post BUP dose | SL BUP (solution)  0, 0.5, 2, 8, 16, 32 mg  (single dose once weekly) | SL BUP attenuated HYD effects in a dose-related manner up to 8 mg; doses higher than 8 mg did not produce a greater effect. |
| Strain 1997 | N=8 | Hydromorphone IM  (0, 9, 18 mg) at 16 hours post BUP dose  [IM BUP: 4, 8, 16 mg] | SL BUP (solution)  8 mg/day | 9 and 18 mg HYD produced typical opioid agonist effects, with little difference between doses, suggesting that BUP maintenance doses higher than 8 mg might be needed to maximize clinical efficacy. |
| Schuh 1999 | N=8 | Hydromorphone IM  (0, 2, 4 mg) | SL BUP (solution)  0, 2, 8 mg/day | 2 mg of SL BUP partially attenuated HYD effects.  8 mg of SL BUP produced nearly complete blockade up to 72 hours. |
| Greenwald 1999 | N=14 | Hydromorphone IM  (0, 4, 8, 16 mg) at 3 hours post BUP dose | SL BUP (solution)  2, 4, 8 mg/day | 8 mg of SL BUP attenuated HYD agonist symptoms relative to a BUP dose of 2 mg. SL BUP did not produce a significant dose-dependent attenuation of HYD reinforcement. There were important individual differences in the efficacy of buprenorphine. |
| Comer 2001 | N=8 | Heroin IV  (0, 6.25, 12.5, 25 mg) | SL BUP (tablet)  8, 16 mg/day | 16 mg of SL BUP reduced heroin self-administration and drug liking compared to 8 mg of SL BUP.  Reinforcing/liking effects of heroin were not fully antagonized by 8-16 mg BUP, suggesting that higher doses might be needed. |
| Strain 2002 | N=6 | Hydromorphone IM  (12 mg) at 1 hour and 25 hours post BUP dose | SL BUP/NLX (tablet)  4/1, 8/2, 16/4, 32/8 mg/day  SL BUP (tablet)  32 mg | The dose of 4/1 mg of SL BUP/NLX was too low to block the effects of HYD although daily doses as high as 32/8 mg failed to completely block these effects. There were no significant differences between 8/2, 16/4 and 32/8 mg. The addition of NLX did not produce additional blockade. |
| Greenwald 2002 | N=14 | Hydromorphone IM  (4, 24 mg) | SL BUP (tablet)  2, 16 mg/day  4, 32 mg/every 2 days | Higher BUP doses significantly decreased HYD 24 mg choice and increased money choice. These beneficial effects were retained when high-dose BUP was administered on alternate days. |
| Greenwald 2003 | N=5 | Hydromorphone IM  (24 mg) | SL BUP (tablet)  0, 2, 16, 32 mg/day | High-dose BUP produced near-maximal MOR occupancy (PET scans) which correlated with higher BUP plasma levels, lower opioid withdrawal symptoms and greater opioid blockade. |
| Comer 2005 | N=7 | Heroin IN  (0, 12.5, 25, 50, 100 mg) | SL BUP/NLX (tablet)  2/0.5, 8/2, 32/8 mg/day | 8/2 and 32/8 mg of SL BUP/NLX performed similarly better compared to 2/0.5 mg with respect to heroin reinforcing & subjective effects. Model-predicted MOR availability decreased by 74%, 83% and 91% at 2/0.5, 8/2 and 32/8 mg of SL BUP/NLX. |
| Correia 2006 | N=8 | Hydromorphone IM  (0, 6, 12 mg) at 2, 26, 50, 74 and 98 hours post BUP dose | SL BUP/NLX (tablet)  8/2, 16/4, 32/8 mg/day | All three BUP doses provided substantial but incomplete blockade against opioid agonist effects for 98 hours. The extent of blockade diminished steadily but modestly over this time |
| Greenwald 2007 | N=10 | Hydromorphone IM  (0, 6, 12, 24 mg) at 4, 28, 52 and 76 hours post BUP dose | SL BUP (tablet)  16 mg/day | MOR occupancy was correlated with BUP plasma concentration and pharmacodynamic effects. MOR occupancy of 50%-60% was required for the control of withdrawal symptoms and opioid blockade. |
| Walsh 2017 | N=47 | Hydromorphone IM  (0, 6, 18 mg) on days 1-3, 4-6, 8-10, and 11-13 | SC weekly depot BUP  24 or 32 mg given on day 0 and day 7 | Opioid blockade was demonstrated on average at all time points for both HYD doses at mean BUP plasma concentrations ranging from 1.5 to 3.8 ng/mL. The variability in scores suggested that a few individuals experienced partial blockade, especially early in treatment. |
| **^*^**This list was built to the best of the authors’ knowledge; some studies could have been missed.  BUP: buprenorphine; HYD: hydromorphone; IM: intramuscular; IN: intranasal; IV: intravenous; MOR: *mu*-opioid receptor; NLX: naloxone; PET: positron emission tomography; SC: subcutaneous; SL: sublingual (transmucosal). | | | | |

**References:**

Bickel WK, et al. Buprenorphine: dose-related blockade of opioid challenge effects in opioid dependent humans. *J Pharmacol Exp Ther*. 1988;247(1):47–53.

Comer SD, et al. Buprenorphine sublingual tablets: effects on IV heroin self-administration by humans. *Psychopharmacology (Berl).* 2001;154(1):28–37.

Comer SD, et al. Buprenorphine/naloxone reduces the reinforcing and subjective effects of heroin in heroin-dependent volunteers. *Psychopharmacology (Berl).* 2005;181(4):664–675.

Correia CJ, et al. Effects associated with double-blind omission of buprenorphine/naloxone over a 98-h period. *Psychopharmacology (Berl).* 2006;189(3):297–306.

Greenwald M, et al. Buprenorphine duration of action: mu-opioid receptor availability and pharmacokinetic and behavioral indices. *Biol Psychiatry*. 2007;61(1):101–110.

Greenwald MK, et al. Effects of buprenorphine maintenance dose on μ-opioid receptor availability, plasma concentrations, and antagonist blockade in heroin-dependent volunteers. *Neuropsychopharmacology*. 2003;28(11):2000–2009.

Greenwald MK, et al. Opioid reinforcement in heroin-dependent volunteers during outpatient buprenorphine maintenance. *Drug Alcohol Depend*. 1999;56(3):191–203.

Greenwald MK, et al. Effects of buprenorphine sublingual tablet maintenance on opioid drug-seeking behavior by humans. *Psychopharmacology (Berl)*. 2002;160(4):344–52.

Jasinski DR, et al. Human pharmacology and abuse potential of the analgesic buprenorphine: a potential agent for treating narcotic addiction. *Arc Gen Psychiatry*. 1978;35(4):501–516.

Mello NK, Mendelson JH. Buprenorphine suppresses heroin use by heroin addicts. *Science*. 1980;207(4431):657–659.

Rosen MI, et al. Buprenorphine: duration of blockade of effects of intramuscular hydromorphone. *Drug Alcohol Depend*. 1994;35(2):141–149.

Schuh KJ, et al. Onset, magnitude and duration of opioid blockade produced by buprenorphine and naltrexone in humans. *Psychopharmacology (Berl)*. 1999;145(2):162–174.

Strain EC, et al. Blockade of hydromorphone effects by buprenorphine/naloxone and buprenorphine. *Psychopharmacology (Berl)*. 2002;159(2):161–166.

Strain EC, et al. The effects of buprenorphine in buprenorphine-maintained volunteers. *Psychopharmacology (Berl)*. 1997;129(4):329–338.

Walsh SL, et al. Effect of buprenorphine weekly depot (CAM2038) and hydromorphone blockade in individuals with opioid use disorder: a randomized clinical trial. *JAMA Psychiatry*. 2017;74(9):894–902.

Walsh SL, et al. Acute administration of buprenorphine in humans: partial agonist and blockade effects. *J Pharmacol Exp Ther*. 1995;274(1):361–372.

Figure S1. Participant Disposition in the Phase 2 Study

342 screened

303 screen failures

- 212 met exclusion criteria
- 78 did not meet inclusion criteria
- 7 withdrew
- 2 failed HYD challenge criteria at Screening
- 2 failed to stabilize on transmucosal buprenorphine
- 1 sponsor decision
- 1 adverse event
- 1 adverse event

39 subjects randomized to HYD challenges

- 39 entered open-label run-in
- 39 received the first dose of BUP-XR 300 mg

38 in intent-to-treat analysis set^‡^

39 in safety analysis set

9 discontinued

- 3 lost to follow-up
- 3 withdrew
- 3 physician decision

30 completed*

HYD: hydromorphone.

*Per protocol, completers were defined as subjects who completed all HYD challenges during Weeks 1-4 of the study.

^‡^ The intent-to-treat analysis set included all subjects who received at least 1 dose of BUP-XR and had at least 1 complete sequence (i.e., 0 mg [placebo], 6 mg and 18 mg HYD in randomised order) of HYD challenges following BUP-XR administration.

Figure S2. Model Predictions vs. Observations for the Number of Hydromorphone Units Earned After Challenge with 6 mg Hydromorphone (a), 18 mg Hydromorphone (b) or Placebo (c) Over the Course of the Phase 2 Study


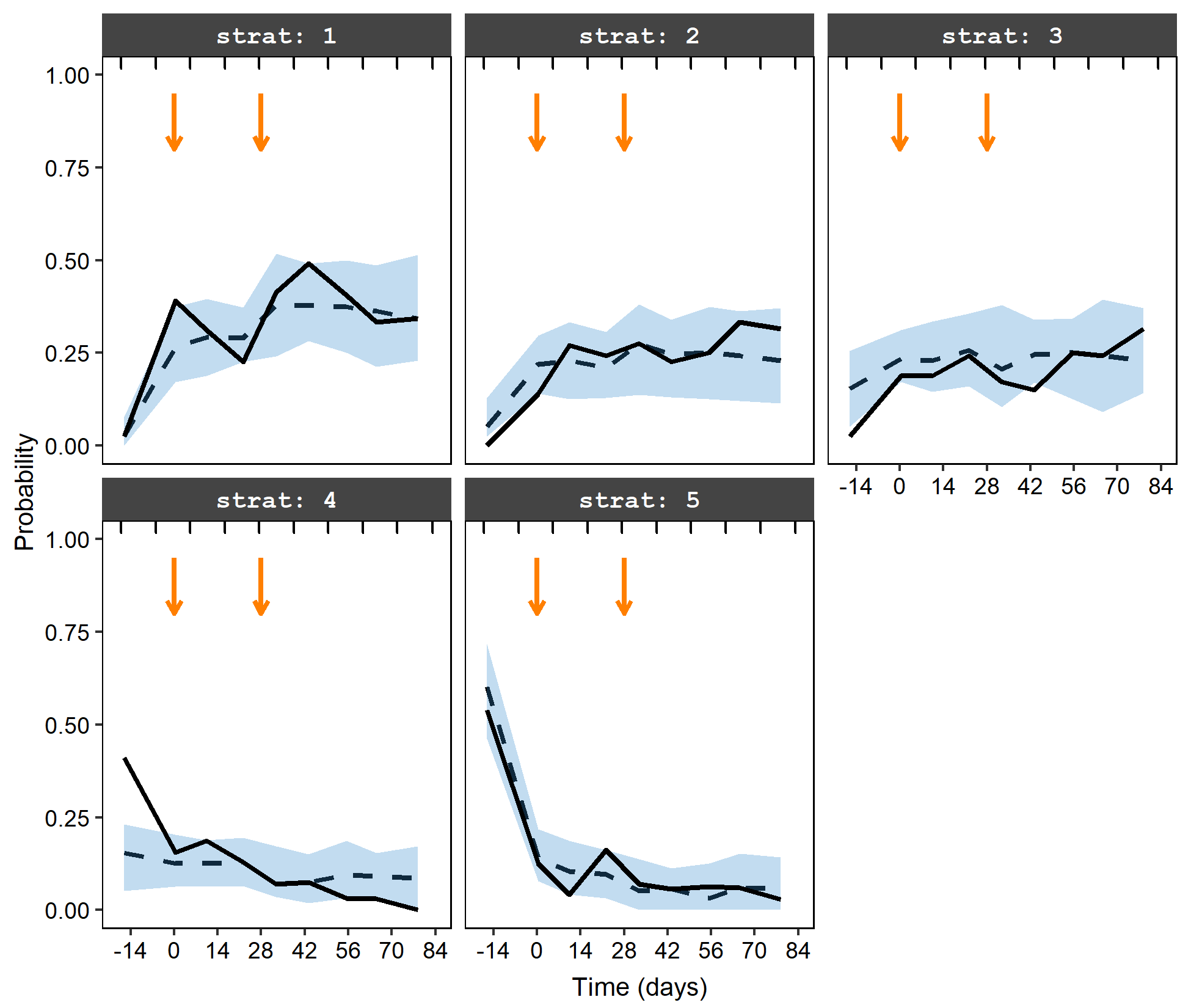


**a**


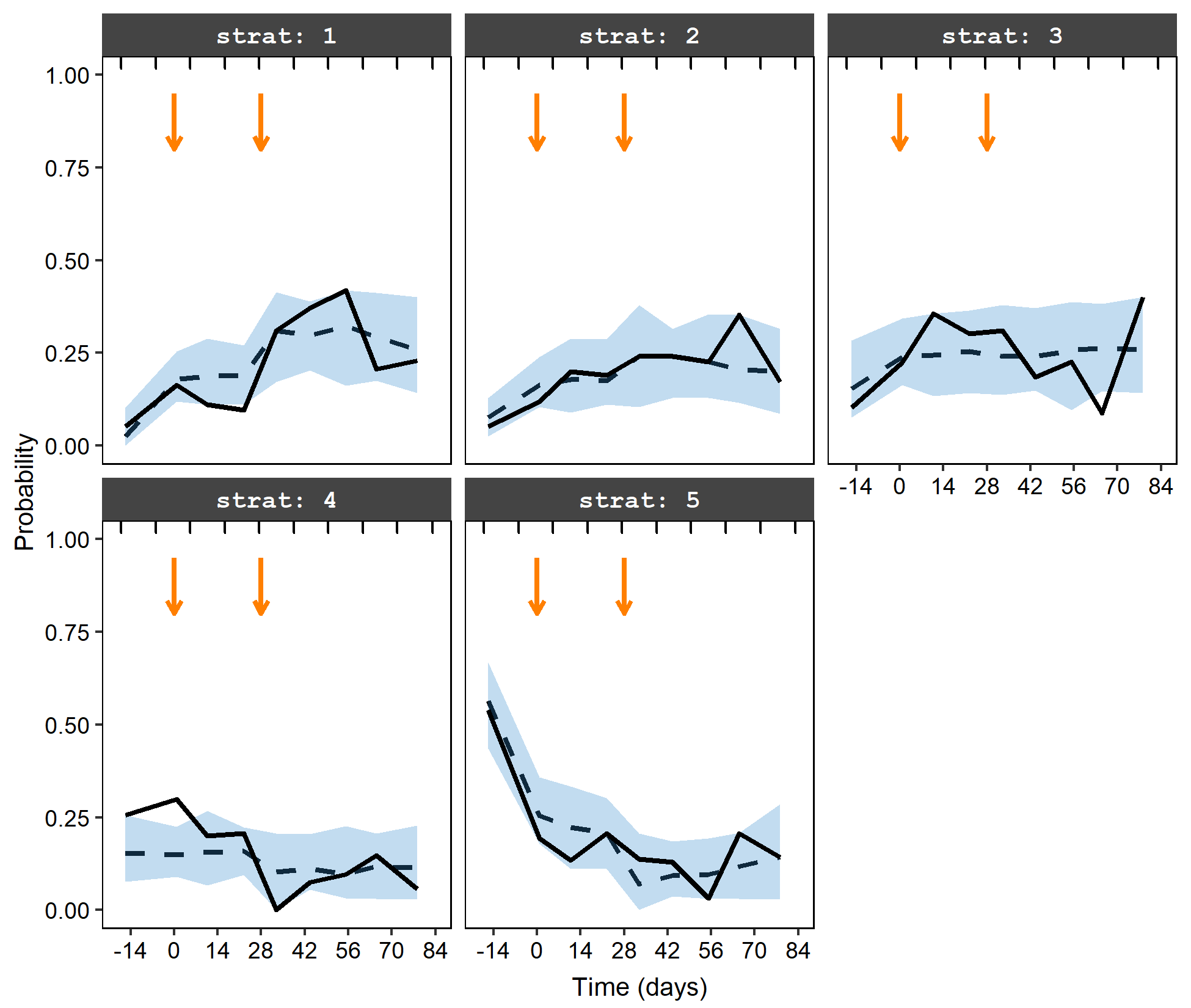


**b**


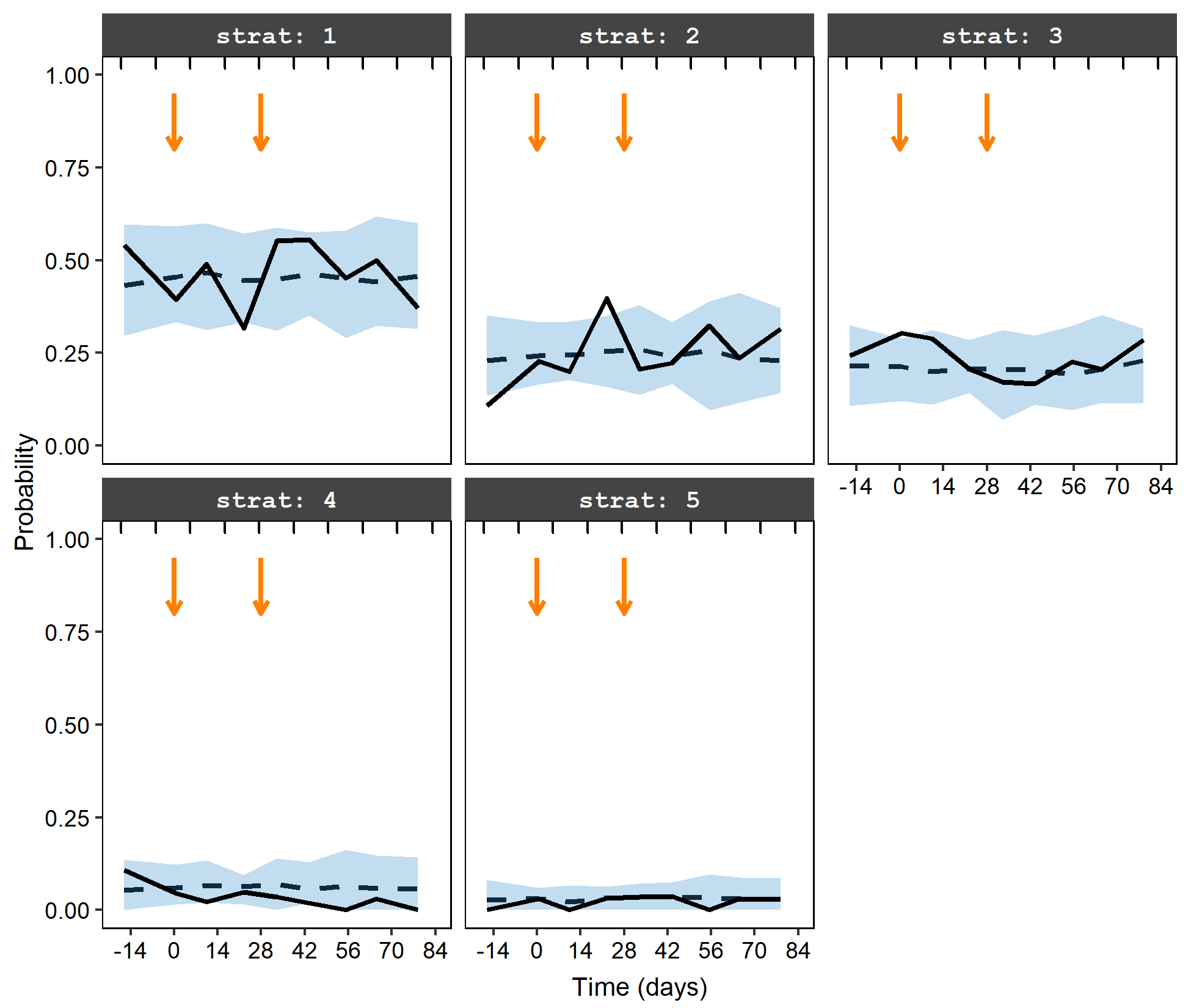


**c**

Model predictions vs. observations for the probability of earning a given number of hydromorphone units were plotted as a function of time (time zero corresponds to administration of the first BUP-XR injection). Data were categorized as follows: 1 (zero hydromorphone unit; subjects only chose money); 2 (1-3 hydromorphone units); 3 (4-6 hydromorphone units); 4 (7-11 hydromorphone units); and 5 (12 hydromorphone units; subjects only chose hydromorphone).

Bold line: observations; dashed line: model predictions; shaded areas: 95% confidence intervals for model predictions. The two vertical arrows represent the two monthly subcutaneous injections of 300 mg BUP-XR.

Figure S3. Participant Disposition in the Phase 3 Study

522 excluded before run-in phase*

160 run-in failures†

1 randomised in error‡

1187 screened*

665 in open-label run-in phase

505 randomised

100 assigned to placebo + IDC

201 assigned to BUP-XR 300/300 mg+ IDC

203 assigned to BUP-XR 300/100 mg+ IDC

34 completed study

100 in full analysis set

99 in full analysis set excluding site 20

89 in per-protocol analysis set

100 in safety analysis set

129 completed study

201 in full analysis set

196 in full analysis set excluding site 20

183 in per-protocol analysis set

201 in safety analysis set

125 completed study

203 in full analysis set

194 in full analysis set excluding site 20

185 in per-protocol analysis set

203 in safety analysis set

66 discontinued

- 12 lost to follow-up
- 18 withdrew consent
- 7 other§
- 18 lack of efficacy
- 2 adverse events
- 3 withdrawal symptoms
- 2 non-compliance with study drug
- 3 investigator withdrawal
- 1 physician decision

72 discontinued

- 23 lost to follow-up
- 21 withdrew consent
- 6 other§
- 5 lack of efficacy
- 10 adverse events¶
- 5 protocol deviation
- 1 withdrawal symptoms
- 1 physician decision

78 discontinued

- 26 lost to follow-up
- 20 withdrew consent
- 17 other§
- 3 lack of efficacy
- 6 adverse events
- 2 protocol deviation
- 1 withdrawal symptoms
- 2 non-compliance with study drug
- 1 investigator withdrawal

Site 20 was closed by the sponsor because of compliance issues. All 15 participants from that site were excluded from efficacy analyses.

*Including 34 participants who met the screening criteria but did not enter the run-in phase.

†Individuals who did not meet randomisation criteria within 7–14 days of dosing with buprenorphine-naloxone sublingual film were considered run-in failures.

‡This participant was randomly assigned to the 300/300 mg group, but did not receive study treatment.

§Discontinuation due to other reasons included: site closed by sponsor (n=9), incarceration (n=7), relocation (n=4), non-compliance with study visits/lost to follow-up type reasons (n=4), participant withdrew (n=3), sponsor request after participant failed to meet eligibility criteria (n=2), and pregnancy (n=1).

¶Including one death.

Figure S4. Mean (±SD) Buprenorphine Plasma Concentration-Time Profiles for the Two Dosing Regimens Evaluated in the Phase 3 Study


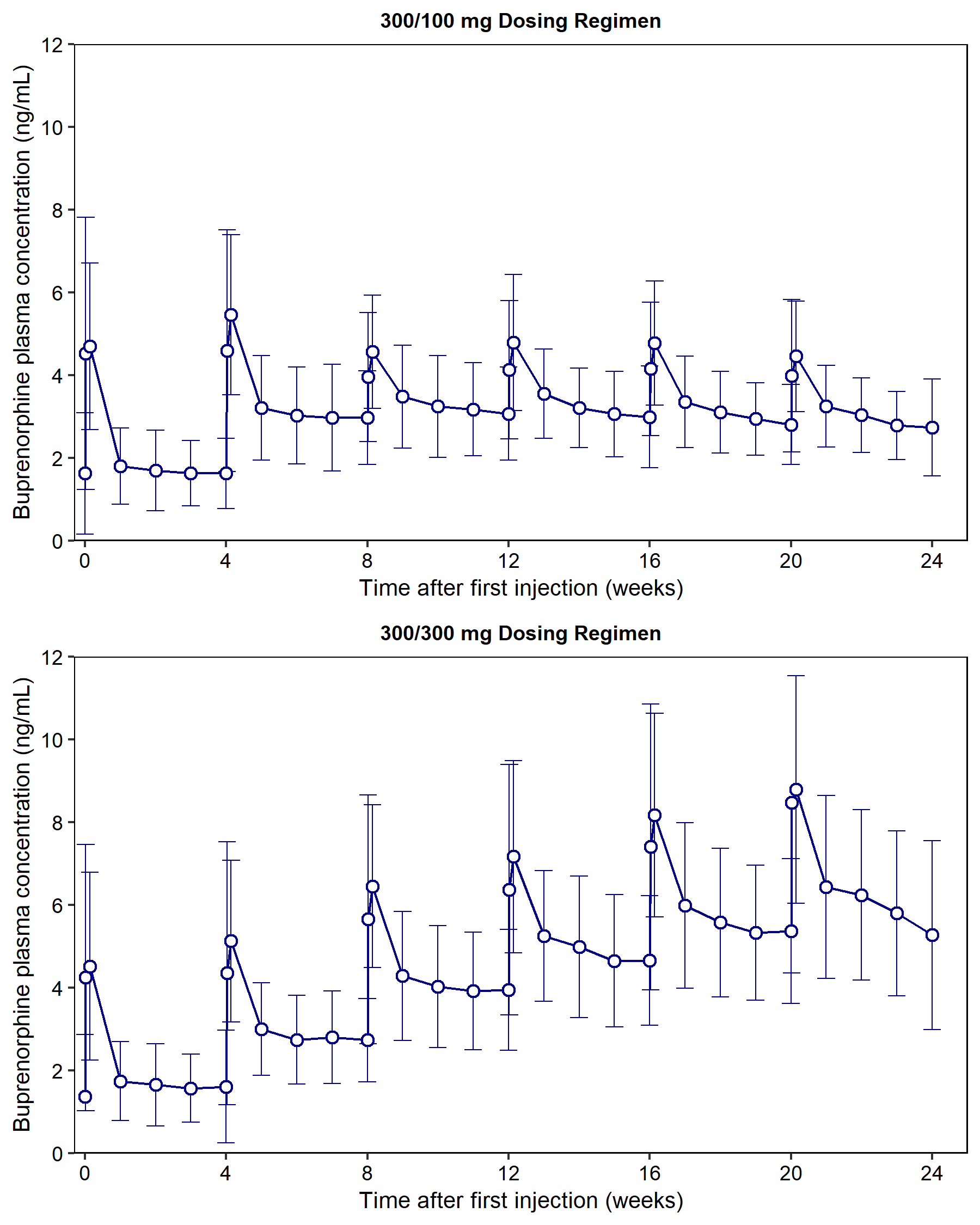


300/100 mg dosing regimen: 2 monthly injections of 300 mg BUP-XR followed by 4 monthly injections of 100 mg BUP-XR (n=203)

300/300 mg dosing regimen: 6 monthly injections of 300 mg BUP-XR (n=201)

SD: standard deviation

Figure S5. Model Predictions vs. Observations for the Number of Participants with Negative Opioid Use (a) and Within Each Level of Opioid Craving (b) for Each Treatment Group of the Phase 3 Study


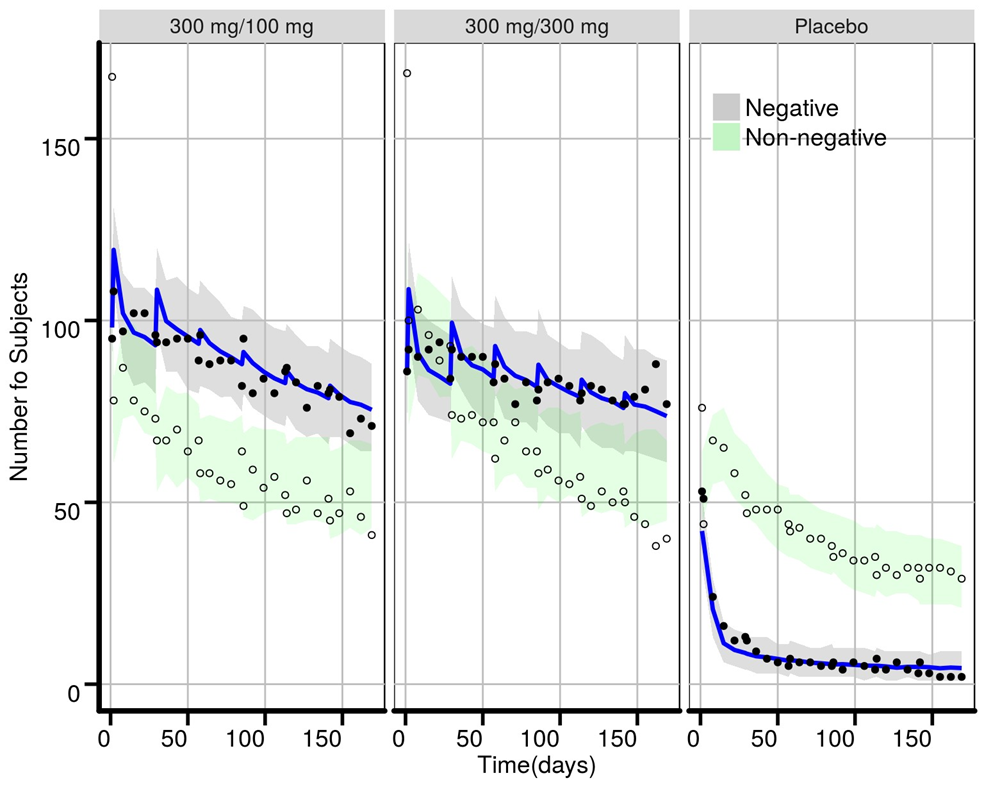


**a**


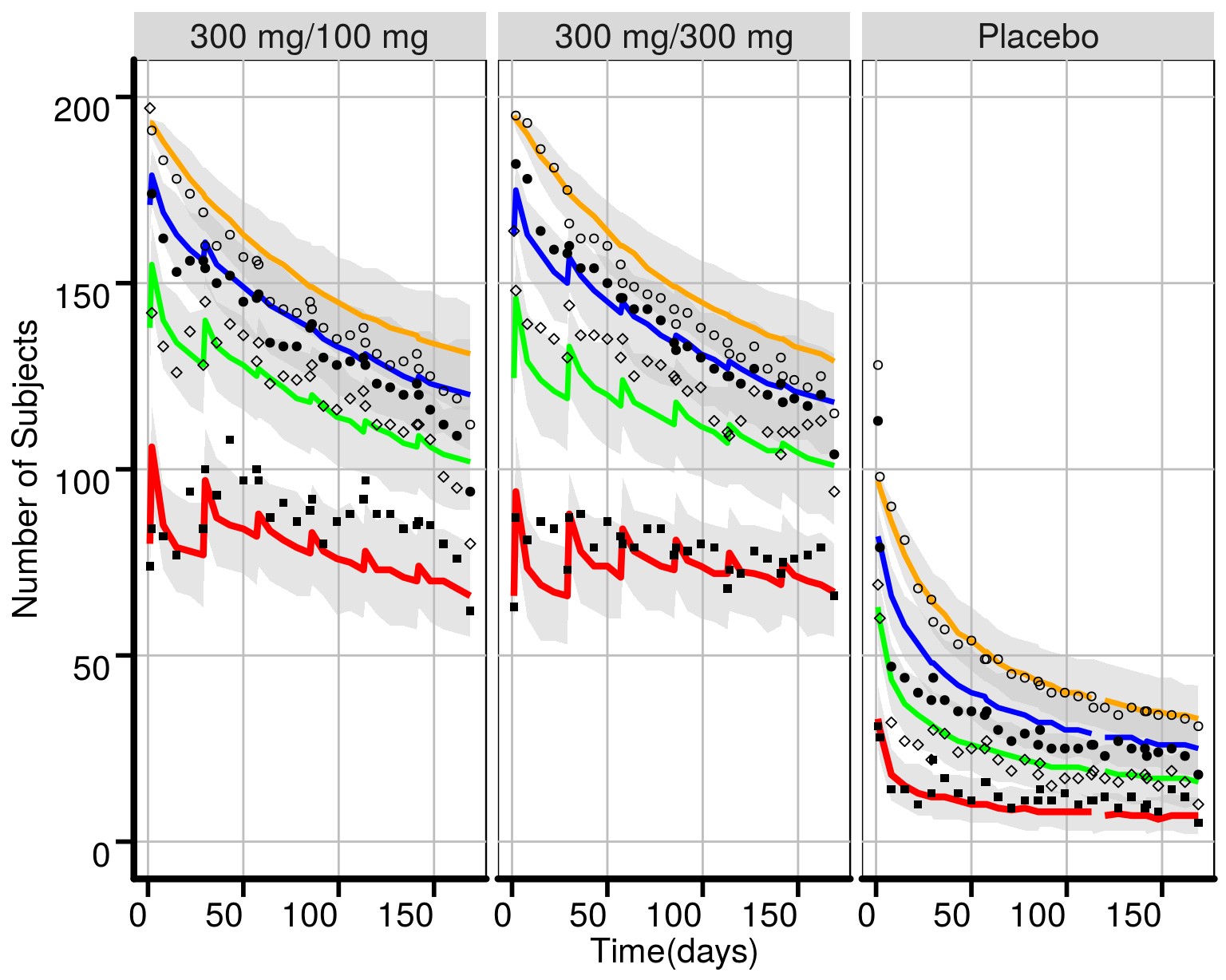


**b**

**a)** Opioid use. Dots represent observations; the blue curve model predictions for negative opioid use; and the shaded areas the 95% confidence region for model predictions.

**b)** Opioid craving. Dots represent observations; the different curves represent model predictions for craving equal to 0 (red), ≤ 5 (green), ≤ 20 (blue) and total number of participants (yellow); and the shaded areas the 95% confidence region for model predictions.

Initial subject numbers at randomisation in each treatment group were 194 (300/100 mg), 196 (300/300 mg), and 99 (placebo).

Figure S6. Model Predictions vs. Observations for Dropout Per Treatment Group in the Phase 3 Study


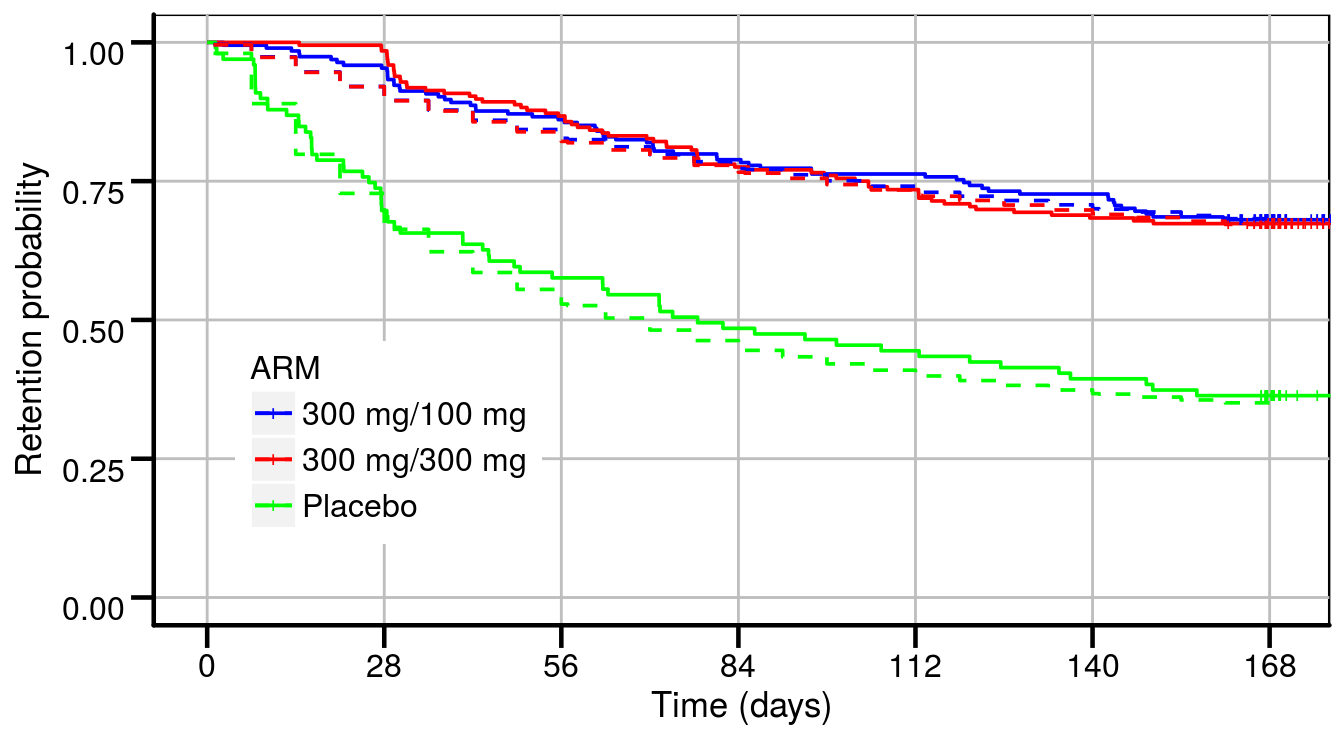


Bold lines: Kaplan Meier curves for observations; Dashed lines: Kaplan Meier curves for model predictions.

Figure S7. Effect of Race on Dropout Rate Per Treatment Group in the Phase 3 Study


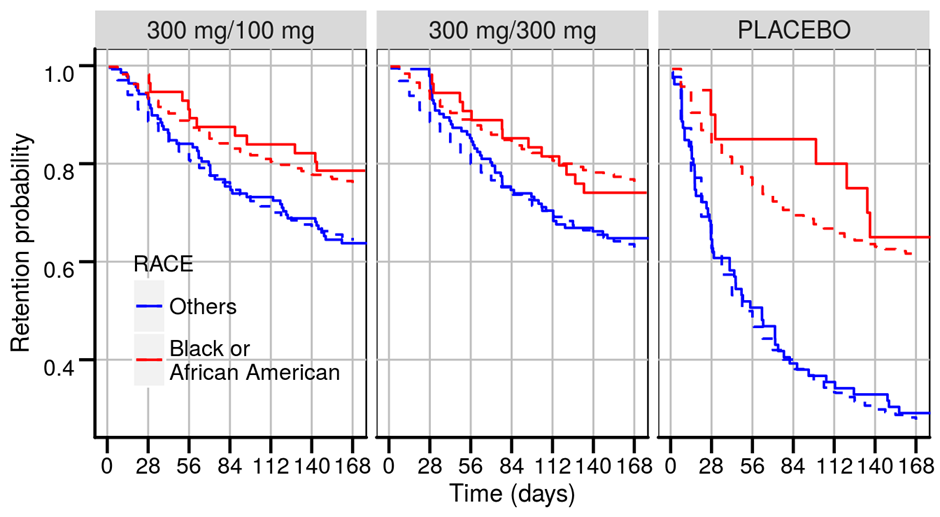


Bold lines: Kaplan Meier curves for observations; Dashed lines: Kaplan Meier curves for model predictions.

Figure S8. Effect of Age on Dropout Rate Per Treatment Group in the Phase 3 Study


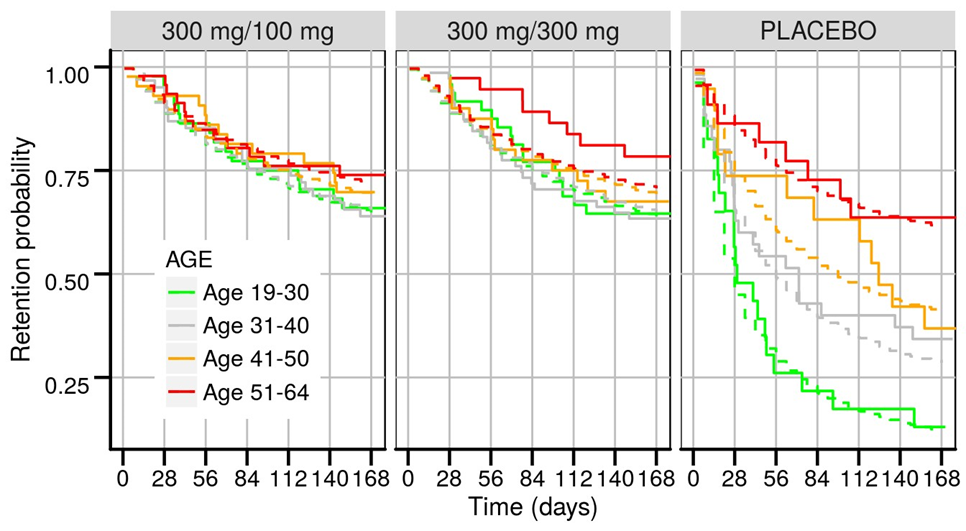


Bold lines: Kaplan Meier curves for observations; Dashed lines: Kaplan Meier curves for model predictions.

Figure S9. Effect of CGI-S on Dropout Rate Per Treatment Group in the Phase 3 Study


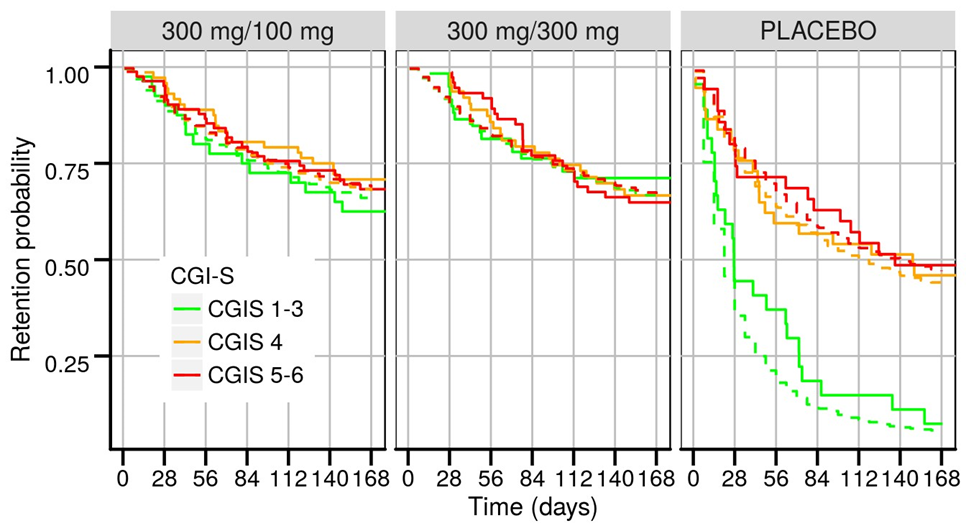


CGI-S: Clinical Global Impression-Severity scale

Bold lines: Kaplan Meier curves for observations; Dashed lines: Kaplan Meier curves for model predictions.

**Population Pharmacokinetic/Pharmacodynamic Model Equations:**

**Modeling of hydromorphone reinforcing effects (Phase 2 study)**

The number of hydromorphone units earned during each session ranged between 0 and 12 and was analysed as an ordinal variable. To limit the number of categories, data were divided into 5 categories ordered from 1 to 5: zero unit (subjects only chose money, $m$=1); 1-3 units ($m$=2); 4-6 units ($m$=3); 7-11 units ($m$=4); and 12 units (subjects only chose hydromorphone, $m$=5). The selection of the categories was data-driven to achieve sufficient proportions of observations in each group.

Data were analysed using mixed-effects logistic regression for ordinal measures. If $Y_{ij}$ denotes the observation in subject $i$ at time $t_{ij}$ ($j=1\ldots n_{i}$), the probability for $Y_{ij}$ to be lower or equal to $m$ ($m=$1, 2, 3 or 4) was expressed as follows:

$$logit\left[ P\left( Y_{ij}\leq m \right) \right]=\alpha_{m}-{BASE}_{HYD}\times\left( 1-fd \right)+\eta_{i}$$

Where $\alpha_{m}$ is the intercept, ${BASE}_{HYD}$ is the effect of the hydromorphone challenge dose in the absence of drug, $fd$is the drug effect, and $\eta_{i}$ is the subject-specific random effect introduced on the intercept. To account for the ordinal nature of the data, $\alpha_{1}<\alpha_{2}<\alpha_{3}<\alpha_{4}$.

A sigmoidal E_max_ model of buprenorphine plasma concentration was selected for $fd$:

$$fd=\frac{E_{max,i}\times{C_{p,ij}}^{\gamma}}{{{EC}_{50,i}}^{\gamma}+{C_{p,ij}}^{\gamma}}$$

Where $E_{max,i}$is the maximal effect (fixed to 1) and ${EC}_{50,i}$ the buprenorphine plasma concentration reaching 50% of maximal effect in subject $i$; $\gamma$ is the Hill coefficient; and $C_{p,ij}$ denotes the buprenorphine plasma concentration in subject $i$ at time $t_{ij}$ predicted by the population pharmacokinetic (PK) model based on empirical Bayes estimates (EBEs).

The probability of observing a score in a given category was formulated as:

$$P\left( Y_{ij}=1 \right)=P\left( Y_{ij}\leq1 \right)$$

$$P\left( Y_{ij}=2 \right)=P\left( Y_{ij}\leq2 \right)-P\left( Y_{ij}\leq1 \right)$$

$$P\left( Y_{ij}=3 \right)=P\left( Y_{ij}\leq3 \right)-P\left( Y_{ij}\leq2 \right)$$

$$P\left( Y_{ij}=4 \right)=P\left( Y_{ij}\leq4 \right)-P\left( Y_{ij}\leq3 \right)$$

$$P\left( Y_{ij}=5 \right)=1-P\left( Y_{ij}\leq4 \right)$$

**Modeling of dropout (Phase 3 study)**

Missing data due to dropout of subjects from the Phase 3 trial were modeled using time-to-event analysis. Dropout was successfully predicted from baseline subject characteristics and recorded measures of efficacy, supporting missing-at-random mechanisms. The probability of a subject dropping out at a particular time was predicted by describing the hazard for dropout. Hazard is the instantaneous risk of dropping out and was modeled for active treatment groups as follows:

$$hazard=\beta_{0 TRT}\times\beta_{1 TRT}^{Craving LOCF}\times\beta_{2}^{RACE}exp\left( -{ke}_{TRT}\times t \right)$$

The model equation for hazard in placebo group was the following:

$$hazard=\beta_{0 PBO}\times\beta_{1 PBO}^{Craving LOCF}\times\beta_{2}^{RACE}\times\left( \frac{Age}{38} \right)^{\beta_{3 PBO}}\times\beta_{4 PBO}^{CGI-S \leq3}\times exp\left( -{ke}_{PBO}\times t \right)$$

where:

- $\beta_{0}$ is the baseline hazard in the active treatment group (TRT) or placebo group (PBO),
- $\beta_{1}^{Craving LOCF}$ accounts for the effect of craving based upon the last recorded observation (last observation carried forward or LOCF) in the active treatment group (TRT) or placebo group (PBO),
- $\beta_{2}^{RACE}$ accounts for the effect of race (African Americans vs. others [mainly White subjects]),
- $\beta_{3 PBO}$ accounts for the effect of age (median value: 38 years) in the placebo group only,
- $\beta_{4 PBO}^{CGI-S \leq3}$ accounts for the effect of disease severity (CGI-S ≤ 3 vs. CGI-S ≥ 4) in the placebo group only,
- $ke$ is the rate constant for the exponential decrease in hazard over time (Gompertz model) in the active treatment group (TRT) or placebo group (PBO).

The whole history of opioid craving records since the beginning of the trial was used to predict survival until the time of dropout or until the end of the trial for subjects completing the study. Survival to a given time ($t_{i}$) was calculated for each subject $i$ as follows:

$$Survival(t_{i})=exp\left( -\int_{0}^{t_{i}} hazard(t)dt \right)$$

**Modeling of opioid use (Phase 3 study)**

Opioid use (composite variable based on urine drug screen and self-report for illicit opioid use) was modeled as a binary variable where values were either 0 (negative opioid use) or 1 (positive opioid use). Only observed data were used for pharmacokinetic/pharmacodynamic modeling and no data were imputed.

Data were analysed using mixed-effects logistic regression. If $Y_{ij}$ denotes the observation in subject $i$ at time $t_{ij}$ ($j=1\ldots n_{i}$), the probability of abstinence (opioid use = 0) was modeled as:

$$logit\left[ P\left( Y_{ij}=0 \right) \right]=\alpha+fd+\eta_{i}$$

$$P\left( Y_{ij}=0 \right)=\frac{exp\left( \alpha+fd+\eta_{i} \right)}{1+exp\left( \alpha+fd+\eta_{i} \right)}$$

Where $\alpha$ is the intercept, $fd$is the drug effect, and $\eta_{i}$ is the subject-specific random effect introduced on the intercept. Consistent with the empirical concentration-response evaluation, an E_max_ model of the buprenorphine plasma concentration was selected for $fd$:

$$fd=\frac{E_{max,i}\times C_{p,ij}}{{EC}_{50,i}+C_{p,ij}}$$

Where $E_{max,i}$is the maximal effect and ${EC}_{50,i}$ the buprenorphine plasma concentration reaching 50% of maximal effect in subject $i$; $C_{p,ij}$ denotes the buprenorphine plasma concentration in subject $i$ at time $t_{ij}$ predicted by the population PK model based on EBEs.

**Modeling of opioid craving (Phase 3 study)**

Opioid craving was categorized into 4 categories ordered from 1 to 4: zero craving ($m$=1); craving 1-5 ($m$=2); craving 6-20 ($m$=3); and craving > 20 ($m$=4). Data were analysed using mixed-effects logistic regression for ordinal measures. If $Y_{ij}$ denotes the observation in subject $i$ at time $t_{ij}$ ($j=1\ldots n_{i}$), the probability for $Y_{ij}$ to be lower or equal to $m$ ($m=$1, 2 or 3) was expressed as follows:

$$logit\left[ P\left( Y_{ij}\leq m \right) \right]=\alpha_{m}+fd+\eta_{i}$$

Where $\alpha_{m}$ is the intercept, $fd$is the drug effect, and $\eta_{i}$ is the subject-specific random effect introduced on the intercept. To account for the ordinal nature of the data, $\alpha_{1}<\alpha_{2}<\alpha_{3}$.

Consistent with the empirical concentration-response evaluation, an E_max_ model of the buprenorphine plasma concentration was selected for $fd$:

$$fd=\frac{E_{max,i}\times C_{p,ij}}{{EC}_{50,i}+C_{p,ij}}$$

Where $E_{max,i}$is the maximal effect and ${EC}_{50,i}$ the buprenorphine plasma concentration reaching 50% of maximal effect in subject $i$; $C_{p,ij}$ denotes the buprenorphine plasma concentration in subject $i$ at time $t_{ij}$ predicted by the population PK model based on EBEs.

The probability of observing a score in a given category was formulated as:

$$P\left( Y_{ij}=1 \right)=P\left( Y_{ij}\leq1 \right)$$

$$P\left( Y_{ij}=2 \right)=P\left( Y_{ij}\leq2 \right)-P\left( Y_{ij}\leq1 \right)$$

$$P\left( Y_{ij}=3 \right)=P\left( Y_{ij}\leq3 \right)-P\left( Y_{ij}\leq2 \right)$$

$$P\left( Y_{ij}=4 \right)=1-P\left( Y_{ij}\leq3 \right)$$
